# Climatic and environmental changes of ~100 thousand years: The mammals from the early Middle Pleistocene sequence of Notarchirico (southern Italy)

**DOI:** 10.1371/journal.pone.0311623

**Published:** 2024-10-23

**Authors:** Beniamino Mecozzi, Alessio Iannucci, Marco Carpentieri, Antonio Pineda, Rivka Rabinovich, Raffaele Sardella, Marie-Hélène Moncel

**Affiliations:** 1 Dipartimento di Scienze della Terra (PaleoFactory lab.), Sapienza Università di Roma, Roma, Italy; 2 Dipartimento di Biologia Ambientale, Sapienza Università di Roma, Roma, Italy; 3 Department of Geosciences, Section of Terrestrial Palaeoclimatology, Eberhard-Karls-University Tübingen, Tübingen, Germany; 4 Dipartimento di Studi Umanistici, Università degli Studi di Ferrara, Ferrara, Italy; 5 UMR 7194 HNHP (MNHN-CNRS-UPVD), Département Homme et Environnement, Muséum National d’Histoire Naturelle, Paris, France; 6 Institut Català de Paleoecologia Humana i Evolució Social (IPHES-CERCA), Tarragona, Spain; 7 National Natural History Collections, Institute of Earth Sciences, Institute of Archaeology, The Hebrew University of Jerusalem, Jerusalem, Israel; Tel Aviv university, ISRAEL

## Abstract

Here we revise all the paleontological sample of Notarchirico, including historical collections and new findings collected during 2016–2023 excavations. Notarchirico is one of the most significant sites for the study of human evolution and terrestrial ecosystem dynamics during the Early-Middle Pleistocene Transition, preserving nearly 100.000 years of environmental and climatic changes constrained between 695 ± 6 ka and 614 ± 12 ka. The deposit yielded the oldest human fossil of the Italian Peninsula, and one of the oldest European evidence of *Homo heidelbergensis*, as well as one of the earliest evidence of bifacial tools in western Europe, commonly associated with the Acheulean techno-complex. Our paleontological results revealed the presence of three different mammal complexes, documenting faunal dynamics in response of climatic driven-changes recognized during the early Middle Pleistocene. The lower complex (levels I2-G) indicates the dominance of wooded spaces, sparse steppes, and the existence of water bodies (lakes or ponds), indicating a deterioration of the fully interglacial conditions recorded during the end of MIS 17; the middle complex (levels G-C) with a low number of mammal remains can be attributed to the glacial conditions of MIS 16; the upper complex (levels B-above α) indicates an improvement in climate, transitioning towards the full interglacial conditions of the of MIS 15. The faunal sample of Notarchirico, based on its firm chronological setting, offers important data for the Biochronological Scheme of European Land Mammals, including one of the oldest records of *Palaeoloxodon antiquus* and *Cervus elaphus* in Europe, *Panthera spelaea* in southwestern Europe, *Dama* cf. *roberti* in Italian Peninsula, and one of the latest occurrences of *Bison schoetensacki* in Europe.

## Introduction

Significant climatic changes characterized the latest Early Pleistocene and the Middle Pleistocene of Europe, representing one of the major events for the evolution of the European terrestrial ecosystems. This period, known as Early–Middle Pleistocene Transition (EMPT, or Middle Pleistocene Revolution) records the shift of the glacial–interglacial climate cycles from 41 ka to approximately 85–125 ka [1, 2], profoundly impacting both vegetation and terrestrial mammal communities in Europe [3–5]. The climatic changes provoked increased seasonality and aridity in the northern Hemisphere, with some of the most severe glacial conditions recognized during the Marine Isotopic Stage (MIS) 16, and MIS 12 [6].

The mammal paleocommunities of the EMPT document major reorganizations and a gradual disappearance of the Villafranchian [7–10]. A severe genetic bottleneck affected human populations during this time [11, 12]. The progressive dispersal of modern taxa and the emergence of the Acheulean techno-cultural complex, generally associated with the dispersal of *Homo heidelbergensis*, is also recognized [7–10, 13–18].

Notarchirico, a site located in southern Italy, has a long sedimentary sequence recording almost 100.000 years, constrained between 695 ± 6 ka and 614 ± 12 ka [16, 19], and plays a relevant role for the study of human evolution and terrestrial ecosystem dynamics during the EMPT in Mediterranean Europe [16, 19–21].

The site was occupied repeatedly, representing a *unicum* within the European context in displaying a very early Acheulean lithic assemblage, with some layers without handaxes [16, 20]. In addition, a hominin fossil (Vn-H1) was found in the upper part of the sequence (level α), represented by the proximal two-thirds of a right femur lacking the proximal epiphyseal region and referred to as an immature individual (possibly a late adolescent) of *Homo heidelbergensis* [21].

Mammal fossil remains have been found along the sequence, but only the sample from the upper levels (A and α) was described in detail by Cassoli et al. [22]. Based on the rich sample from the upper levels, the Authors [22] proposed a new Faunal Unit (FU; biochronological units initially defined on the basis of the Italian fossil record and subsequently widely adopted in western Europe), with an age intermediate between that of the FUs of Isernia and Fontana Ranuccio, but the fauna was then attributed to the Isernia FU [23–25].

Here, the fossil remains found during the historical excavations coordinated by Virginia Ginetta Chiappella and Marcello Piperno, stored at the National Archeological Museum of Venosa “Mario Torelli” and the Museum of Civilizations (Rome, central Italy) are revised, as well as those still *in situ* preserved at the Paleolithic Park of Notarchirico (Venosa, southern Italy) and the new findings collected during 2016–2023 excavations are also here collectively presented for the first time. The results provide valuable data on the changes of the faunal record of Notarchirico sequence and of the faunal changes it documents over time, and, more in general, towards the understanding of the terrestrial ecosystem evolution in the Mediterranean Europe during the EMPT.

## Notarchirico

### History of excavations

**Fig 1:**
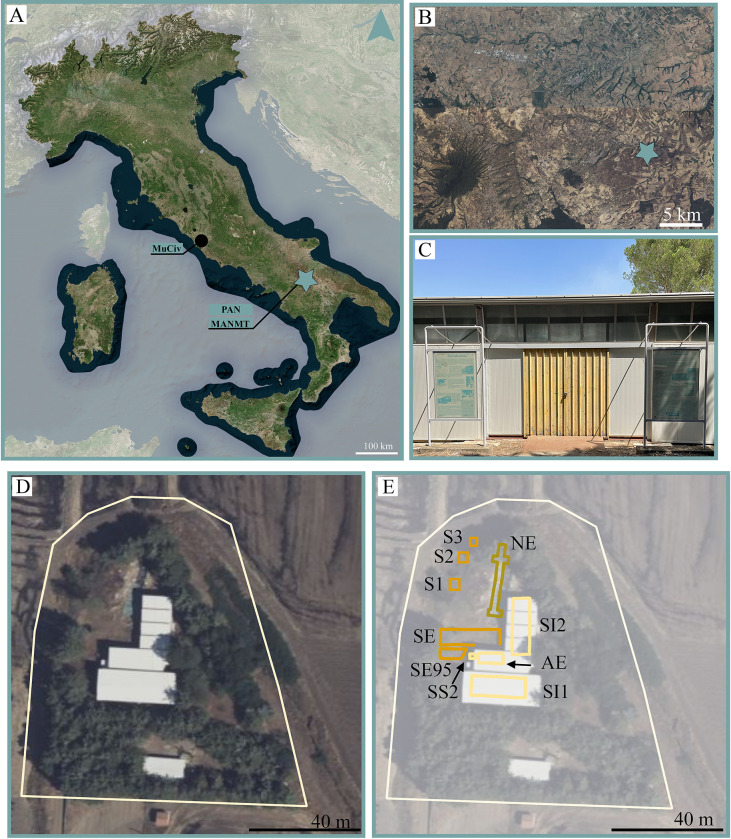
The site of Notarchirico (Venosa, southern Italy). Its geographic location (the star indicates the position of the site; A-B); the external view of the building within the Paleolithic Park of Notarchirico (C); the extension of the Paleolithic Park of Notarchirico and the position of historical and new excavation trenches (D-E). Abbrevations: SE–Scavo Esterno; SI1 –Scavo interno 1; SI2 –Scavo interno 2; AE–Area dell’Elefante; SE95 –Scavo Esterno 1995; SS2 –Sondaggio di salvataggio 2; S1 –Sondaggio 1; S2 –Sondaggio 2; S3 –Sondaggio 3. Images A-B visualised and elaborated within QGIS 3.38 software with “QuickMapServices” plugin https://nextgis.com/blog/quickmapservices/). Image C allowed by the agreement of the Italian Ministry of Culture and the Museo Parco Archeologico Melfi e Venosa.

The Venosa Basin represents a significant area for the investigation of human evolution and the influence of the climatic fluctuations on the terrestrial ecosystems during the EMPT (Fig 1). The region hosts numerous deposits containing abundant archeological and paleontological remains within extensive sedimentary sequences. Geochemical analyses and radiometric dating of materials from the Vulture Volcano (Fig 1) yielded significant chronological data, placing the majority of these sites between 700 ka and 500 ka [16, 26–28].

Several of these sites have been discovered and excavated during the first half of 20^th^ century, as Loreto and Terranera [29]. The first fieldwork at Notarchirico was coordinated by Virginia Ginetta Chiappella, who conducted a stratigraphical survey in 1956 [29]. Chiappella recovered a rich archeological and paleontological record from a borehole whose exact location was unfortunately not provided, dividing the sample in 24 artificial levels, each of 10 cm, for a total depth of 240 cm. She noted the presence of five pebble levels at depths of 70 cm, 100 cm, 130 cm, 170 cm and 240 cm [29].

Systematic investigations in Notarchirico truly began in 1979, when members of the Italian Institute of Human Palaeontology (IsIPU), including Aldo Giacomo Segre, Eugenia Segre-Naldini, and Italo Biddittu, conducted archaeological, geological, and paleontological surveys in the Venosa Basin [29, 30].

The first three excavation campaigns (1980, 1981, 1984) were coordinated by Marcello Piperno and E. Segre-Naldini, covering 156 m^2^, and resulted in the recovery of a rich archeological and paleontological sample. The investigated area was then named “Scavo Esterno” (SE) (meaning “outside excavation” in Italian), following the building of a structure thought to accommodate, protect and musealize *in situ* the remarkable palaeosurfaces of the “inside excavation” and their fossil and lithic material (Fig 1).

**Fig 2:**
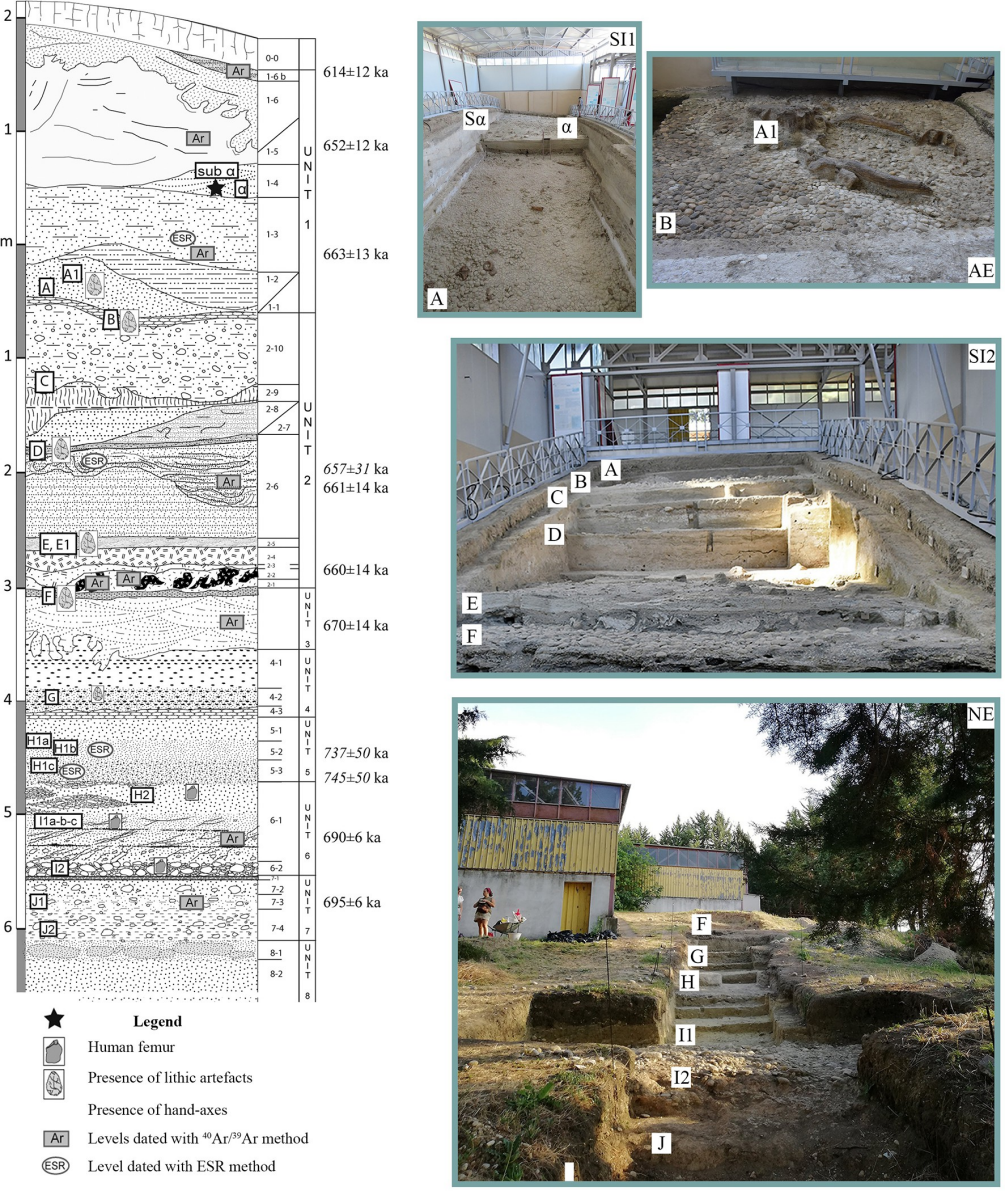
Stratigraphic succession of Notarchirico and its excavated trenches during historical and new fieldwork excavations. (see Fig 1 for the position of trenches). Image allowed by the agreement of the Italian Ministry of Culture and the Museo Parco Archeologico Melfi e Venosa.

From 1985 to 1995, the fieldworks have been focused in three different areas preserved inside the building, called “Scavo interno 1” (SI1) (meaning “inside excavation” in Italian), “Scavo interno 2” (SI2) and “Area dell’Elefante” (AE; meaning “Elephant Area” in Italian) (Figs 1 and 2).SI1 covers an area of 131 m^2^ where fossil and lithic material from the levels above α, α, A and B were exposed; SI2 encompasses 120 m^2^ of a trench containing levels from A to F; finally, AE represents a lateral discontinuity of 24 m^2^ exposing fossils found in the level A and A1 [29]. An additional area, located outside the building (referred to as SE95), of level D (3 m^2^), was excavated in 1995. Furthermore, an area of 4 m^2^ preserved elephant bone in level C (“Sondaggio di Salvataggio 2”; SS2) was exposed during the construction of the building. The excavation at the bottom of the sequence was limited to boreholes located outside the building, named “Sondaggio 1” (S1), “Sondaggio 2” (S2) and “Sondaggio 3” (S3). S1 identified the level G (16 m^2^), S2 revealed the level H (9 m^2^), and S3 exposed the level I (4 m^2^; without separation between I1 and I2 recognized during new excavations). They had revealed possible older levels with both fossil and lithic material.

Since 2016, new excavations took place outside the building on the hill slope, starting from the base of the previously studied sequence (level F; Figs 1 and 2). A long trench of more than 30 m has been opened, exposing a series of levels, called F, G, H, I1, I2 and J, with faunal and lithic remains. The results of the numerous multidisciplinary studies have been published in numerous papers [16, 20, 21, 31–35].

In both the old and new excavations, the sediments were sieved to recover small-sized remains.

### Chronostratigraphic context

New series of dating allowed revising the chronology of the Notarchirico sequence excavated during the 1980s and 1990s, placing it between 670 ± 4 ka to 614 ± 12 ka [16, 19, 20] (Fig 2; Table 1). This chronology falls mainly within the full glacial conditions of MIS 16, with only a partial overlap at the beginning of MIS 15.

**Table 1:** Stratigraphical, chronological, archeological and palentological data from Notarchirico. *from [16, 26]; **chronology ESR ages in italic. Non italic 40AR/39AR; *** from [29] layers α to F, new excavations 2016–2023 layers F to J; ****Extracted from [29]; *****[35].

Lithostratigraphic Unit*	Archeo Unit	Chronology	Characteristics	m^2^ excavated	Nb artefacts***	Nb fauna remains
1,4	α	612 ± 5ka	Archeosurface α. 50 cm. Reworked tephras.	Around 70 m^2^	949	827
		650 ± 4ka	Sandy and clayey deposits			
1,1	A-A1-B	658 ± 9ka	Archeodurface B. Pavement of pebbles.	A 120m^2^	A 316	606
			At the top of subunit 2.10- Volcanic sands.	A1 24 m^2^	A1 41	23
				B 133 m^2^	B 351	48
2,9	C		Brown clayey deposit. Pavement of pebbles. Archeosurface C at the top of sub-unit 2.9.	20 m^2^	78	61
			30 cm			
2,6	D	*657 ± 31ka*	Volcanic sands with clayey beds and carbonates.	25 m^2^	290	113
		661 ± 4ka	Coarse sediments for archeosurface D			
2,5	E		Volcanic sands with carbonates and reworked tephras.	42 m^2^	E 309	179
			Archeosurface E with small pebbles at the top of sub-unit 2.5.		E1 285	84
3	F	659 ± 4ka 658 ± 6ka 668 ± 6ka	Cross-bedded volcano-derived and non-volcanic sands 20 cm.	Piperno 19 m^2^	19	9
			Archeosurface F. Pavement of pebbles.	New excavations. 16 m^2^	411	315
			Black volcanic sands 20 cm			
4.1–4.2	G		Brown with small gravel 1m Dark-grey volcanic sands	15 m^2^	543	333
			Archeosurface G. 30 cm			
5,3	H	737 ± 50ka** 745 ± 50ka	Silty-sandy deposit. Sandier and oxidized with a few micro-beds of dark minerals 30 cm	10 m^2^	75	83
			Dispersed archeological material			
6.1–6.2	I	690 ± 6ka	Dispersed archeological material I1	I1 18 m^2^	I1 451	1129
			Archeosurface I2. Dense accumulation of cobbles and smaller elements with limestones pebbles and a few fine-grained sandstone cobbles and flint nodules 10–15 cm	I2 26 m^2^	I2 171	515
7.1–7.3			Tuffaceous sub-unit 3 cm			
			Coarse yellow sands with a few cobbles 15 cm Tephra-derived coarse sands with some cobbles 10 cm			
7,4	J	690 ± 6ka	Cobbles in a clayish volcano-derived matrix 30 cm	4 m^2^	8	4
8.1–8.2			Light-grey sand and micro-breccia			
			Coarse yellow sands			

The ^40^Ar/^39^Ar results date thin levels of tephra fallouts and reworked tephras between the archaeological levels of the bottom of the sequence (levels J to F), providing ages between 695 and 670 ka. Thus, the bottom of the sequence is framed between 695.2 ± 6.2 ka and 690.3 ± 5.8 ka, which corresponds paleoclimatically to the end of MIS 17.

Three ESR datings also confirmed an early Middle Pleistocene age for Notarchirico [16, 19, 20] (Fig 2; Table 1).

Between 10 to 26 m^2^ were open outside the building, keeping the same stratigraphic levels identified in previous excavations.

The sedimentary succession is made of volcanoclastic sediments reworked by the alluvial environment (Table 1). Each deposit corresponds to relatively short periods of accumulation and re-equilibrium after volcanic inputs and before animal and hominin passages.

Levels I2, F, D, C, B and A are dense accumulations of pebbles and cobbles, pavements remain of lakeshores, including material on and into the pavement (archeosurfaces). The other levels record material among encrusted pebbles and coarse deposits or sandy and clayey sediments filling water channels (levels H and E). Sediments covering the pebbles often attest low-energy fluvial sedimentation and regular inputs of volcanic material. The detailed presentation of the sequence describes series of geological units and sub-units in relation to the Monte Vulture volcano eruption were detailed and synthetized by several authors [16, 19, 29].

### Archeological and taphonomic context

Systematic maps of horizontal and vertical spatial distribution of the material have indicated palimpsests of occupations [20]. Some dense clusters of fauna and lithic material are visible without clear accumulation of some categories of material and specific orientation of the material. Double patina and smooth faces on some parts of lithic pieces attest repeated passages of hominins and re-use of material over a long time. Material on and into the pavement indicate recurrent phases of occupation with possible reworking of pieces by hominins among the bed of pebbles-cobbles. The cutting edges are however rather fresh suggesting limited crushing of the tools by animal and hominin passages even patina indicates that pieces were not buried fast [20]. Evidence of anthropogenic modification of faunal remains by hominins groups are scarce throughout the whole sequence of Notarchirico. The only exception is the AE, which was defended as one of the paradigmatic examples that evidenced the butchering practices and cognitive abilities of Middle Pleistocene hominins in western Europe [36–38]. However, the recent revision of the archeo-paleontological materials and their stratigraphic position in the AE has recently questioned the evidence in supporting the anthropic butchery of the elephant carcass [35].

Apart from the recent studies [20, 35], the only level in which taphonomic analyses were carried out is the level α, in which unequivocal evidence of anthropic exploitation of cervids and bovids were identified through some percussion notches on limb bones [39]. It has been proposed that the lack of evidence could be due to the poor preservation of the bone surfaces, which are heavily damaged as consequence of edaphic processes, weathering effects, or hydraulic action, since some indirect evidences, such as use-wear analysis, suggest the processing of animal-derived resources [20, 39].

### History of paleontological research

The first description of mammals found in the sedimentary succession of Notarchirico was published in 1999, within the book dedicated to the history of excavations and geological, archaeological, and paleontological discoveries from the excavation activities of 1980–1995, coordinated by M. Piperno.

V.G. Chiappella coordinated the first paleontological and archeological survey at Notarchirico in 1956. Unfortunately, there are no information available regarding the exact position of her borehole, thus impeding direct correlations with well-defined stratigraphy of the site. The schematic log realized by Chiappella was only mentioned by [29], which, however, remains unpublished. According to the Author, Chiappella identified the presence of five distinct pebble levels at depths of 70 cm, 100 cm, 130 cm, 170 cm and 240 cm. The long sequence at Notarchirico comprises various pebble levels, named I2, F, D, C, B and A from bottom to top. Chiappella reported a distance of 170 cm between the first and the last pebble levels, while 5 m approximately separate the level I2 from the level B.

The authors also reported a general presence of mammals found in the sequence, with a focus on the AE area where a partial cranium, the jaw, teeth and some postcranial remains referred to as a single young-adult male of *Palaeoloxodon antiquus* (previously assigned to genus *Elephas*) were found in the level A and putatively associated to some stone tools [37, 38]. The AE was interpreted as a butchering site and was restudied by [36], who reinforced this interpretation, i.e., that the AE reflected a single event of processing of an elephant carcass. As aforementioned, the AE is still *in situ* preserved and the entire paleosurface is exposed to visitors. The paleontological and archeological findings, as well as their stratigraphical context have been reevaluated by [35]. The results rejected the interpretation of the AE accumulation as results of a butchery of a single elephant carcass, since the material derives from at least two diverse and unrelated depositional events.

The primary paleontological report was the description of the mammals found in the levels A and α [22]. Additionally, the authors proposed Notarchirico fauna as a new Faunal Unit, but this proposal did not receive significant consensus.

The cranium of *Bison shoetensacki* from the level D was described by [40], who also listed other mammal remains found in the sequence.

The results of the recent excavations were initially synthesized by [16], wherein the preliminary faunal list of the lower part of sequence (levels I2-F) was published for the first time. Based on the findings collected between 2020 and 2022, the taxonomical attributions of mammals were further refined in [20].

Drawing from the paleontological material obtained from the new excavations, Mecozzi et al. [34] described a partial ulna of *Macaca* sp., providing the first evidence of this species at Notarchirico.

In 1985, a shaft of a fossil human femur was discovered in level α [29]. This bone was initially studied by Belli et al. [41] that assigned the femur to an adult female, with morphological affinities to *Homo erectus*. Recently, this fossil (Vn-H1), a proximal two-thirds of a right femur, was attributed to an immature individual of *Homo heidelbergensis* [21].

Finally, a lion metatarsal was identified from layer A, representing the earliest confirmed presence of *Panthera spelaea* in southwestern Europe known to date [33].

## Material & methods

Here, we present the osteological analyses on the large mammal fauna from both the historical collections and the new excavations recovered at Notarchirico.

Mammal remains taxonomically identified in this work from historical fieldwork activities are stored in different institutions (Table 2).

**Table 2:** History of excavations at Notarchirico.

Hosting institution	Numer of fossil remains	Campaigns	Director	Levels
Museum of Civilizations*	87	1956	V. Chiapella	Not defined
Museum of Civilizations*	949	1980–1995	M. Piperno and. E. Segre-Naldini	above α, α, A, B, C, D, E, I
Paleolithic Park of Notarchirico	123	1980–1995	M. Piperno and. E. Segre-Naldini	above α, α, A, A1, B, C, D, E
National Archeological Museum of Venosa “Mario Torelli”	87	1980–1995	M. Piperno and. E. Segre-Naldini	above α, α, A, D
National Archeological Museum of Venosa “Mario Torelli”	315	2016–2023	M.-H. Moncel	F, G, H, I1, I2

*formerly Museo nazionale preistorico etnografico “Luigi Pigorini”

Taxonomic and skeletal element identifications made in this study are based on the reference collection of the PaleoFactory Laboratory, Department of Earth Sciences, Sapienza University of Rome.

Mammal taxonomical identifications are also supported by the study of other late Early to Middle Pleistocene material from European sites.

Measurements of the cranial, dental, and postcranial fossils were taken following von den Driesch [42] and Sorbelli et al. [43] with a digital caliper to the nearest 0.1 mm (S1 Table).

For the identification of *Bison*, the DDW-DEW index was also calculated following Delpech [44] ([DDW/DEW]x100). Bivariate plots of metapodial measurements of selected bovine taxa of Europe include comparative of *Bison* (*Eobison*) *degiulii* from Capena and Pietrafitta [45], Pirro Nord [45, 46], Apollonia [47], *Bison menneri* from Untermassfeld [47]; *Bison schoetensacki* from Cal Guardiola and Vallparadís Estació [43], Durfort [48], Süssenborn [47]; Mauer [49], La Fage [50], Loreto [51], Ponte Galeria [52]; *Bison priscus* from Bugbrook, Barrington and Waterhall Farm [47], Taubach [47], Petralona Cave [47]; *Bos primigenius* from Ilford [53], Castel di Guido [54], Malagrotta [55].

The Canis was compared with the data of *Canis mosbachensis* from Ceré [56], this work, and Soave (this work) and extant *Canis lupus* from Italian Peninsula [56].

The beavers were compared with *Castor fiber* from [57–61], and *Castor plicidens* from [62].

## Results

**Fig 3:**
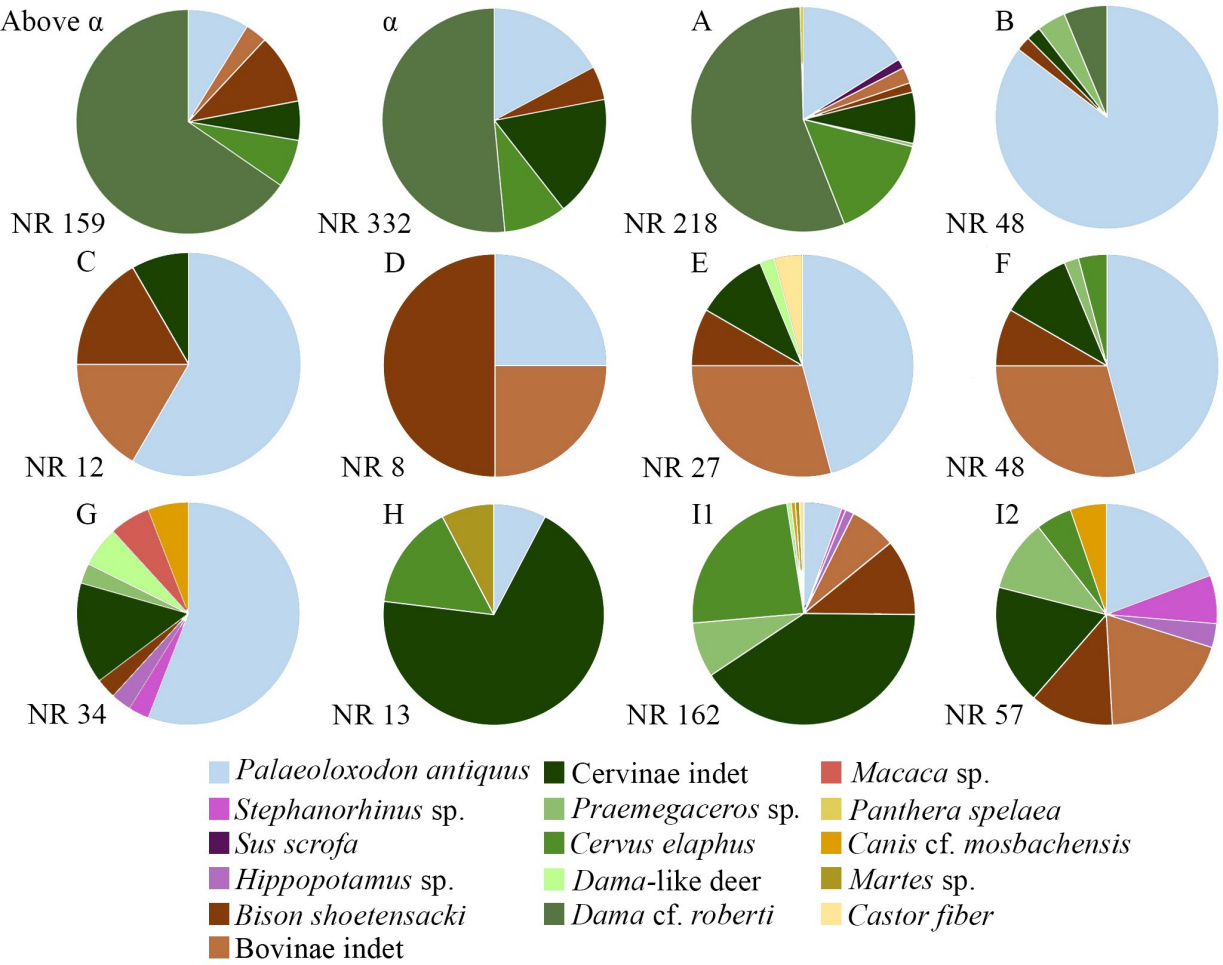
Percentage of the number of the mammal remains taxonomically identified in this work from Notarchirico.

**Fig 4:**
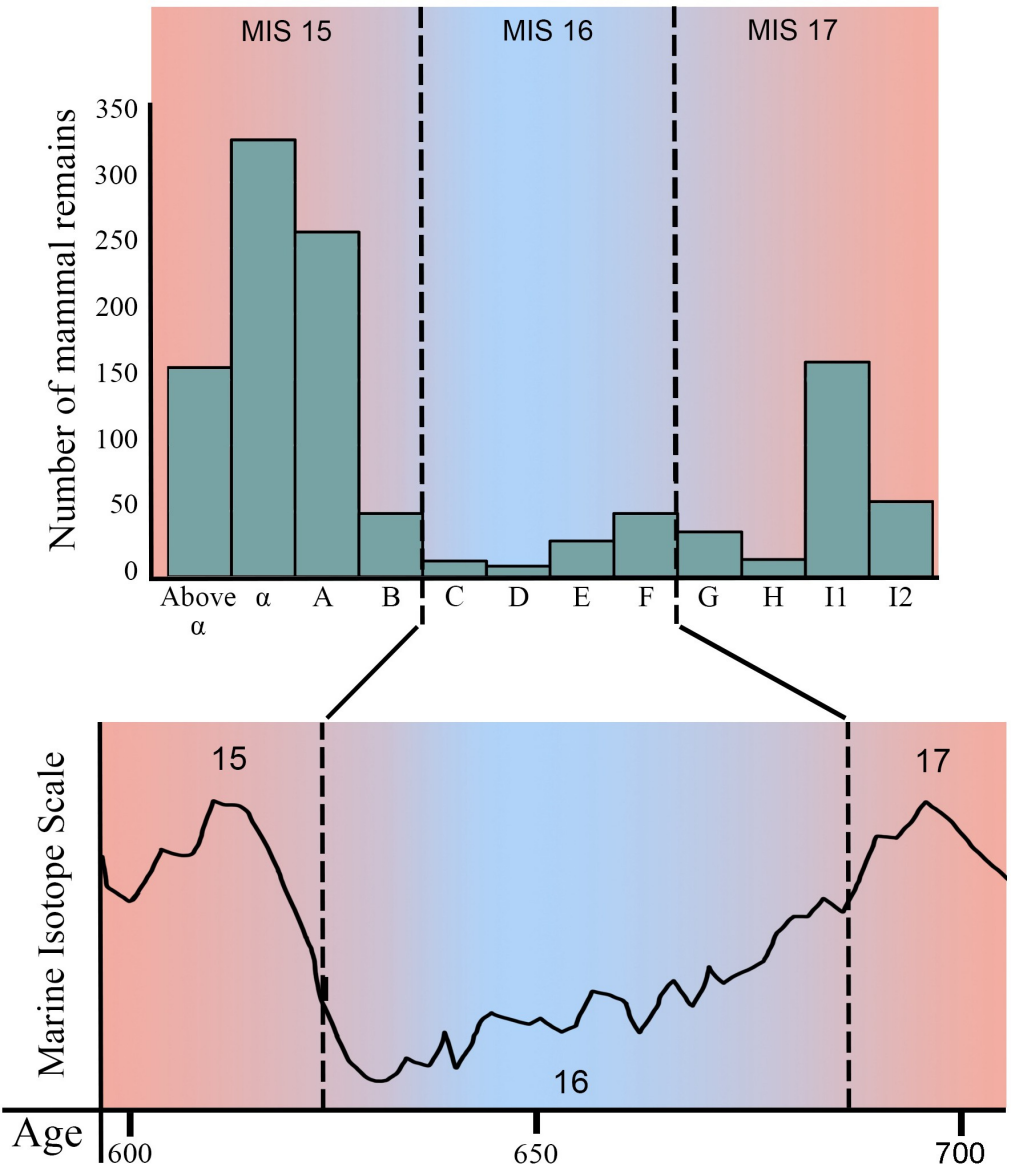
Stratigraphic distribution of mammal remains taxonomically identified in this work from Notarchirico and their attribution according to the chronostratigraphical setting of the site.

The data regarding the number of mammal remains studied in this work and their stratigraphic distribution are reported in Table 3 and Figs 3 and 4.

**Table 3:** Number of taxonomically identified mammal remains from Notarchirico.

Levels Species	above α	α	A	A-α	A1	B	C	D	E	F	G	H	I1	I2	I
*Palaeoloxodon antiquus*	14	57	73	4		41	7	2	3	22	19	1	9	11	6
*Stephanorhinus* sp.											1		1	4	1
*Sus scrofa*			3												
*Hippopotamus* sp.											1		2	2	
Bovinae indet	5		5				2	2	5	14			11	11	
*Bison schoetensacki*	16	16	3	1		1	2	4	9	4	1		18	7	11
Cervinae indet	9	58	21	97	4	1	1		7	5	5	9	66	10	2
*Praemegaceros* sp.			1	16		2				1	1		13	6	1
*Cervus elaphus*	11	30	33	1						2		2	39	3	
Dama-like deer									2		2		1		3
*Dama* cf. *roberti*	104	171	122	158	7	3									
*Macaca* sp.											2				
*Pantera spelaea*			1												
*Canis* cf. *mosbachensis*											2		1	3	
*Martes* sp.												1	1		
*Castor fiber*									1				1		
Total	159	332	262	277	11	48	12	8	27	48	34	13	163	57	24

The majority of the fossil remains has been collected from the top of the sequence, from the levels A, α, and above α (Table 3). Material from the mid part of the sequence (levels C-H) is quite rare (Table 3). The levels at the bottom of succession are relatively rich, especially the level I1.

Cervids are the best represented group in several levels of the sequence, I2, I1 H, above A, α, and above α (Fig 3). Also, *Palaeoloxodon antiquus* is well documented in the deposit, especially in the levels G-B (Fig 3). The other groups are scarcely represented throughout the whole succession, especially carnivorans and primates.

As for the borehole performed by V.G. Chiappella, the fossil sample consists of 87 mammal remains identified at a taxonomic level. She divided the in 24 artificial levels of 10 cm of depth, and reported the stratigraphic position of the archeological and paleontological findings directly on them (Table 4). The cuts 6, 7 and 8 are the richest (40 remains), while the others include sporadic findings. Cervids are the best represented group, with 56 remains attributed to *Dama*-like deer (Table 4). Few fossils are attributed to *Palaeoloxodon antiquus*, with four remains found in the cut 21. The only fossil of carnivoran is a partial canid humerus, which we assigned to *Canis* cf. *mosbachensis*, found in the cut 19, already identified by [37].

**Table 4:** Number of taxonomically identified mammal remains from the borehole coordinated by Virginia Ginetta Chiappella at Notarchirico.

Species	Stratigrpahic cuts																					
	1	2	3	4	5	6	7	8	9	12	13	14	15	16	17	18	19	20	21	24	9–11	5–12
*Palaeoloxodon antiquus*		1							1						1		1		4			
Bovinae indet								2	1											1		
*Bison schoetensacki*			1													1	1	1				
Cervinae indet										2		1	1		1						2	2
*Dama*-like deer	1	2		2	2	15	12	10			1		1	1			1				4	4
*Cervus elaphus*					1			1		1	1											1
*Canis* cf. *mosbachensis*																	1					
Total	1	3	1	2	3	15	12	13	2	3	2	1	2	1	2	1	4	1	4	1	6	7

### Elephantidae

**Fig 5:**
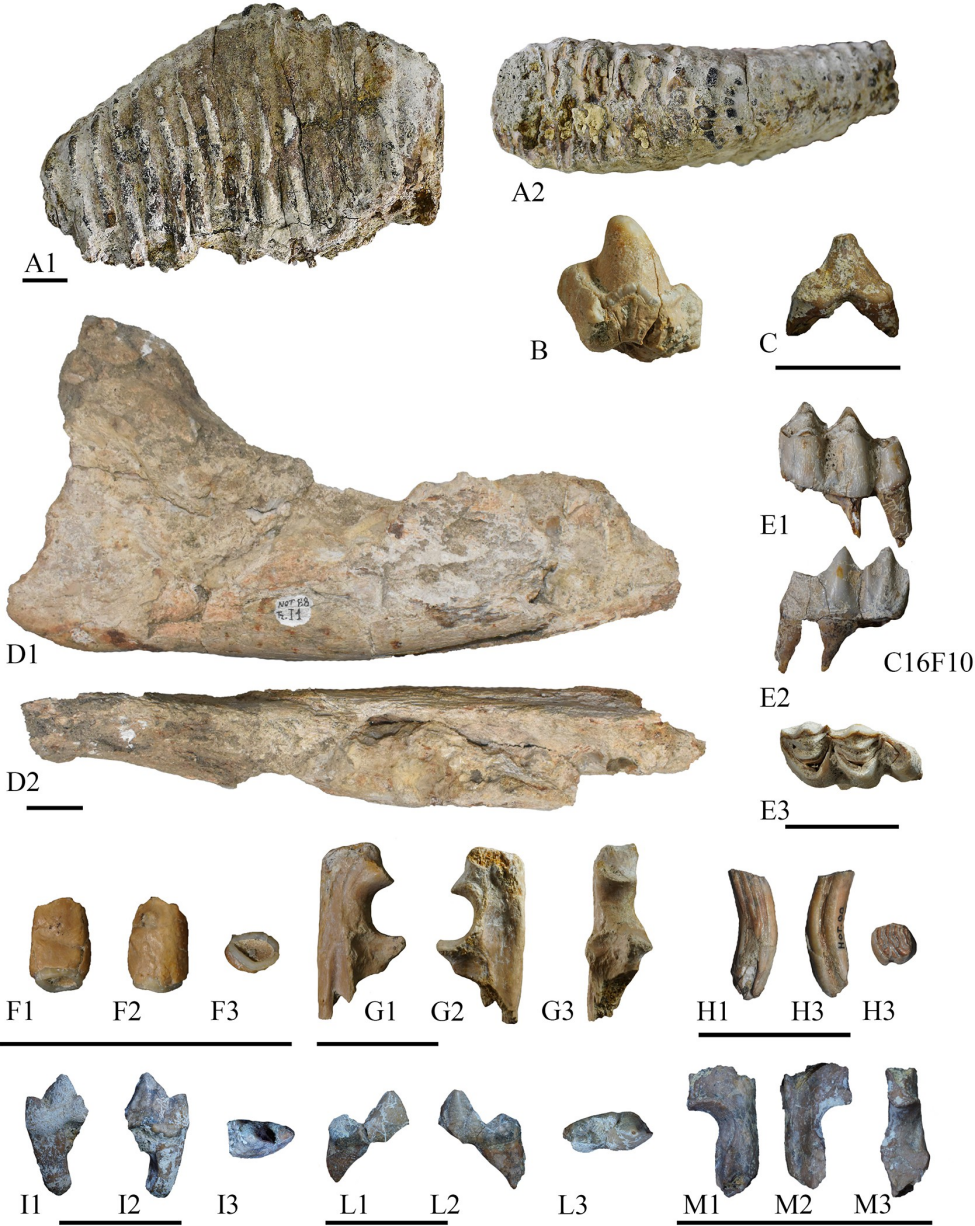
Fossil remains from Notarchirico. A– Z16-37, a right lower third molar of *Palaeloxodon antiquus* in labial (a1) and occlusal (A2) views; B– A22-10, fragment of molar of *Hippopotamus* sp. from the level G; C– A35-SN, right lower second deciduous of *Hippopotamus* sp. from the level I1 in labial view; D–NOT.88-SN, right hemimandible of *Stephanorhinus* sp. from the level I1 in labial (D1) and occlusal (D2) views; E–left lower third molar of *Cervus elaphus* in labial (E1), lingual (E2) and occlusal (E3) views; F– D23-NC1, right deciduous upper canine of *Macaca* sp. in lingual (F1), labial (F2) and occlusal (F3) views; G–B24GNC, proximal right ulna of *Macaca* sp. in medial (G1), lateral (G2) and anterior (G3) views; H–NOT-SN, a right lower third molar of *Castor fiber* in labial (H1), lingual (H2) and occlusal (H3) views; I– F36-NC, a left lower first molar of *Canis* sp. from the level I2 in labial (I1), lingual (I2) and occlusal (I3) views; L– E37-NC2, a right lower first molar of *Canis* sp. from the level I2 in labial (I1), lingual (I2) and occlusal (I3) views; M–B27NC1, proximal left ulna of *Martes* sp. in medial (G1), lateral (G2) and anterior (G3) views. Scale bar = 3 cm. Image allowed by the agreement of the Italian Ministry of Culture and the Museo Parco Archeologico Melfi e Venosa.

Among the megaherbivores, the elephants are one of the best represented groups and the only recovered in all levels (Figs 3 and 5; Table 3). Fossils of this group are generally found isolated and included mainly teeth. The AE is the exception, since the remains recovered (n = 38) were mostly referred to a single young-adult male elephant [35, 36, 38]. Dental remains display several features considered typical of *Palaeoloxodon antiquus*: they are hypsodont and generally possess a high number of laminae, a high lamellar frequency, reduced enamel thickness and a few developed cementum [63–65]. The tusks, well preserved in the AE and in the level C, are long and slightly curved, as typical to *Palaeoloxodon antiquus*.

#### Rhinocerotidae

Rhinoceroses remains are sporadic and found only in the lower part of the sequence (Fig 3; Table 3). During the historical excavations, a hemimandible without teeth was found from the level I, and referred to as *Stephanorhinus* sp. [29] (Fig 5). Several fragments of teeth have been recovered during the new excavations, but lacking morphologies useful for a specific taxonomic attribution. In addition, a carpal bone and a tarsal bone were collected from the levels I1 and G, respectively. As discussed by [66], the postcranial bones of Plio-Pleistocene rhinoceroses possess a wide variability with several polymorphic characters and a strong morphological affinity among taxa. Therefore, the material is attributed to *Stephanorhinus* sp.

#### Suidae

No new suid remain has been recovered during the recent exaction activities, nor newly identified after the revision of the old collections. Only three remains from the level A are attributed to *Sus scrofa* (Fig 3; Table 3). These are a fragment of zygomatic bone, a left tarsal bone and a fragmentary right tibia, as previously described by [22].

#### Hippopotamidae

Hippopotamuses are quite rare and found only in the lower levels (Fig 3; Table 3). The sample includes fragments of teeth and a lower premolar deciduous tooth (Fig 5). The two Quaternary species of hippopotamuses are mainly recognizable based on cranial features [67–69]. The material from level I2, I1 and G were previously studied and attributed to *Hippopotamus* [70]. As the remains are of poor for taxonomical identification we follow their attribution.

#### Bovidae

The bovinae material was found in all levels, except level H (Fig 3; Table 3). The sample mainly consists of fragmentary postcranial bones and isolated teeth, and of few isolated fragmentary horn cores and only a partial cranium collected from the level D.

**Fig 6:**
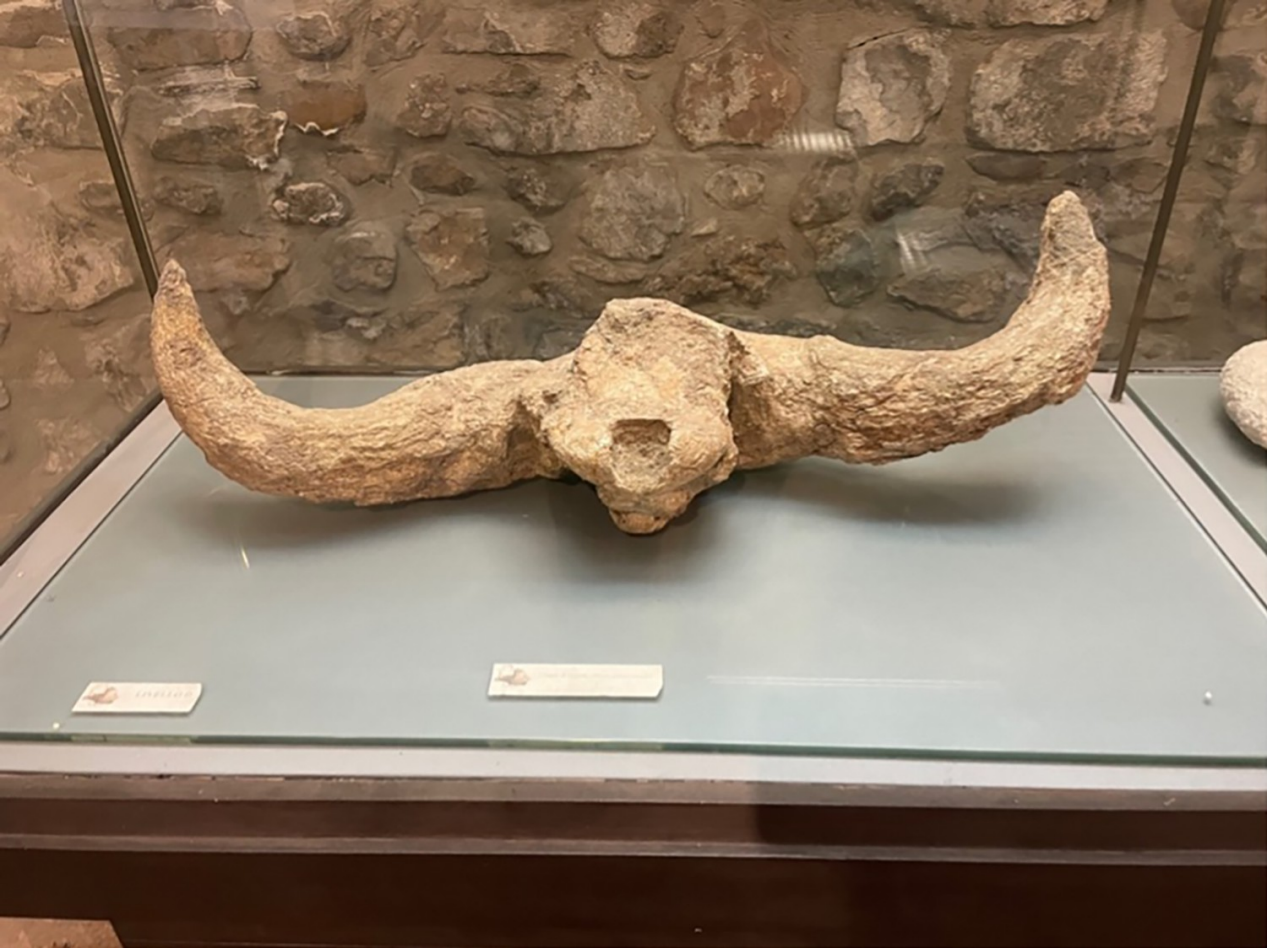
Cranium not. 1995 LIV D of *Bison shoetensacki* from Notarchirico, currently exposed at Museo Archeological Nazionale “Mario Torelli” of Venosa.

This cranium, Not. 1995 LIV D, was ascribed by [40] to *Bison shoetensacki*. The specimen, currently exposed at National Archeological Museum of Venosa “Mario Torelli”, preserves the horn cores, the occipital and the frontal (Fig 6). The cranium is slightly deformed due to the taphonomic process. The horn cores are short and stout and circular at their base, exhibiting a single bend with torsion upward. The pedicle is short and the horn cores emerge laterally forming a near perpendicular angle with the sagittal axis of the cranium. These features are typical of *B*. *schoetensacki* [43, 45].

**Fig 7:**
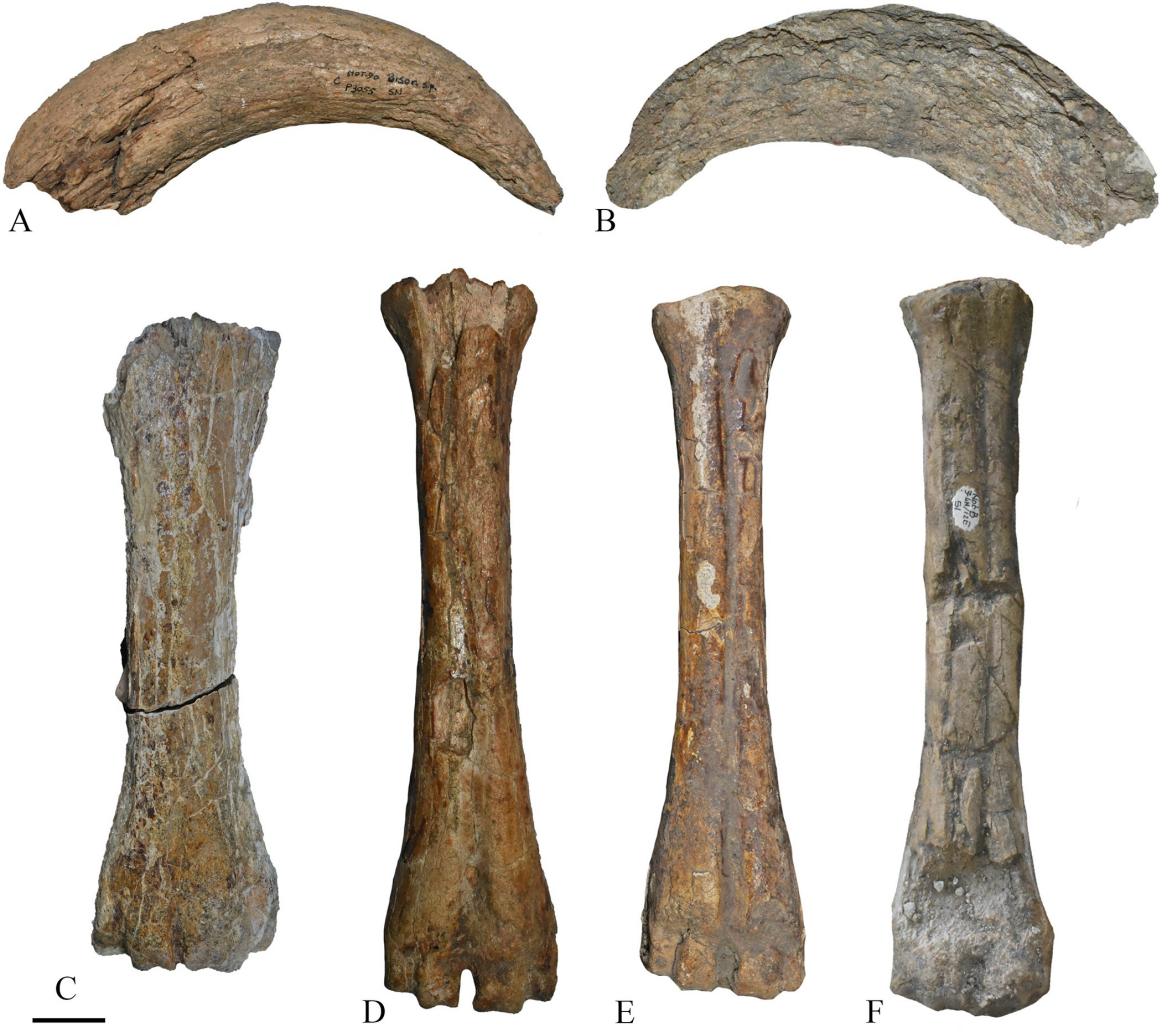
Fossil remains of *Bison shoetensacki* from Notarchirico. A–P3055, left horn core from the level C; B– 858, right horn core from the level C; C– B35-49, left mecarpal from the level I2; D–P3069, left metatarsal form the level I1; E– 2W9S1, right metatarsal from the level alpha; F– 3-4N/12E51, right metatarsal from the level B. Scale bar 3 cm. Image allowed by the agreement of the Italian Ministry of Culture and the Museo Parco Archeologico Melfi e Venosa.

Cassoli et al. [22] reported the presence of Bovinae cfr. *Bos primigenius*, *Bos primigenius*, *Bison* sp. and *Bison schoetensacki* from the levels A and α. In particular, a distal fragment of metatarsal from the level A was ascribed to *Bos primigenius*, while a distal fragment of metatarsal, a fragmentary right calcaneus, a fragmentary right naviculocuboid were attributed to Bovinae cfr. *Bos primigenius*. In the level α, a left metatarsal, a distal fragment of metatarsal, a fragment of distal metacarpal were referred as to *Bos primigenius*, while a right upper third molar, four mandible fragments of the condyle portion, a distal fragment of left scapula, a proximal fragment of right humerus, a proximal fragment of right metatarsal, a distal fragment of metatarsal, a fragmentary left calcaneus and a second phalange were ascribed to Bovinae cfr. *Bos primigenius*. These remains are preserved at Museum of Civilizations and National Archeological Museum of Venosa “Mario Torelli”, and were revised herein (Fig 7).

The upper and lower dental remains show several typical bisontine features: a trapezoidal section of the collar, a straight mesostyle, converging buccal and lingual edges, a swelling at the begins of enamel just above the neck, short ectostylids with an inverted V-shaped profile [43, 71, 72].

Bisontine morphologies are also observed in the postcranial remains, especially in metapodials, which are considered the most useful bones for bovine specific attributions to species [43, 71]. The metapodials of Notarchirico possess a few curved medial and lateral margins in anterior and posterior view, parallel medial and lateral intercondylar crests and the distal width at the tubercle level is larger or roughly equal than condyle one (Fig 7). An additional useful taxonomic feature is a deep and obtuse groove that separates the calcaneal facet from the central tarsal facet on the posterior side of astragalus.

In order to distinguish *Bos* and *Bison*, the DDW-DEW index for metacarpals, introduced by Delpch [44] was also calculated. The value of the two specimens of Notarchirico is 97.3 for 71-C36 from the level I2 and 96.7 for NOT88-SN from the level B, falling in the range of *Bison schoetensacki*, while *Bos primigenius* has lower values (see [43]).

**Fig 8:**
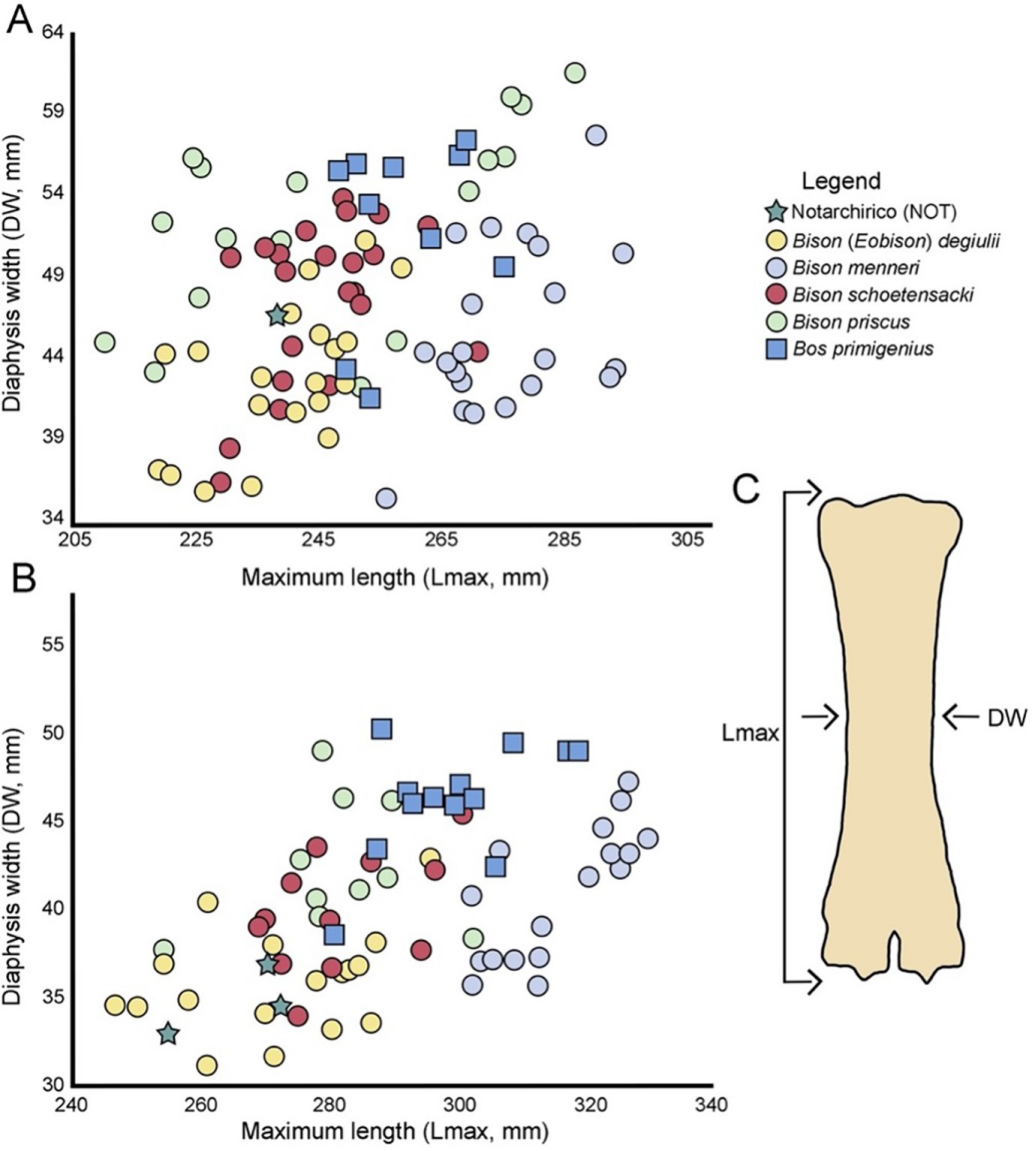
Plot of metacarpal (A) and metatarsal (B) of European bovines from late Early to Middle Pleistocene.

Metarcapal measurements of Notarchirico falls within the range of *Bison schoetensacki* and *Bison* (*Eobison*) *degiulii* (Fig 8), occupying the left part of the graph. *Bison priscus* and *Bos primigenius* generally show larger values of width of the diaphysis (DW) when compared with other species, while *Bison menneri* possesses slender bones. In the metatarsal, the same pattern can be observed, with the specimens from Notarchirico that are among the smallest specimens of the considered sample (Fig 8).

The overall morphology of cranial, dental and postcranial remains of Notarchirico is clearly indicative of *Bison schoetensacki* [43, 71, 73], while the badly preserved and too fragmented fossils are attributed to Bovinae indet (Table 3).

#### Cervidae

Cervids are the most represented group at Notarchirico (Fig 3; Table 3).

Remains of large-sized cervids has been found in almost all levels of the sequence.

Material from historical fieldwork has been ascribed to *Megaceroides* sp. (= *Praemegaceros* sp.; [22, 37]). The sample consists of isolated postcranial bones of poor taxonomical value.

**Fig 9:**
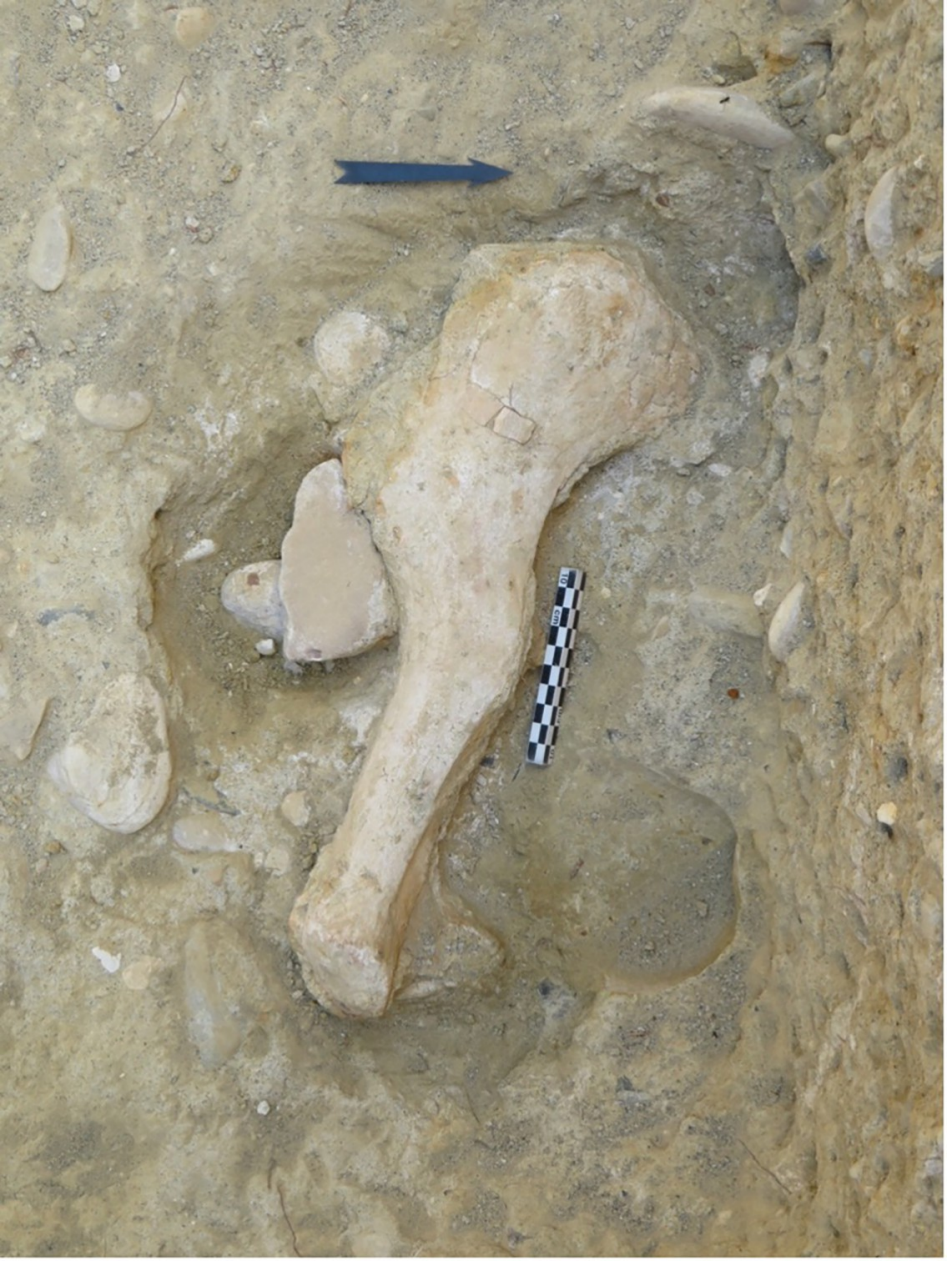
The B33-239 antler from Notarchirico ascribed to *Praemegaceros* sp. during excavations. Image allowed by the agreement of the Italian Ministry of Culture and the Museo Parco Archeologico Melfi e Venosa.

During the new excavations, a partial antler, two fragmentary hemimandibles, several isolated teeth and postcranial elements were found. The antler B33-239 preserves the burr and part of the beam (Fig 9). The section of the burr is quite circular and its plane is slightly inclined with respect to the beam. On the dorsal side, the outer tine is broken a few centimeters above the beam and opened just above the burr. At approximately 10 cm from the outer tine, there is trace of an additional tine (which would be the anterior tine) on the cranial side, but this is totally broken at its base. The dorsal margin of the upper portion of the beam tends to broaden as does the anterior margin, which would have likely resulted in an elongated palmation. These morphologies are generally observed in the genus *Praemegaceros*, strongly resembling *Praemegaceros solilhacus* [74–76].

The red deer, *Cervus elaphus*, is mainly represented by fragmentary postcranial bones and isolated dental remains, found in all levels. Dental remains have several morphological traits that are diagnostic at a specific level: bifurcate entostyle and well-developed buccal cingulum in the upper molars; presence of molarization (the metaconid and entoconid form a closed lingual wall) in the P_4_, in buccal view; protoconid and entoconid connected in lower molars; absence of the step between the lingual walls of second and third lobes in M_3_, in buccal view. These features are indicative of *Cervus elaphus* [77, 78].

**Fig 10:**
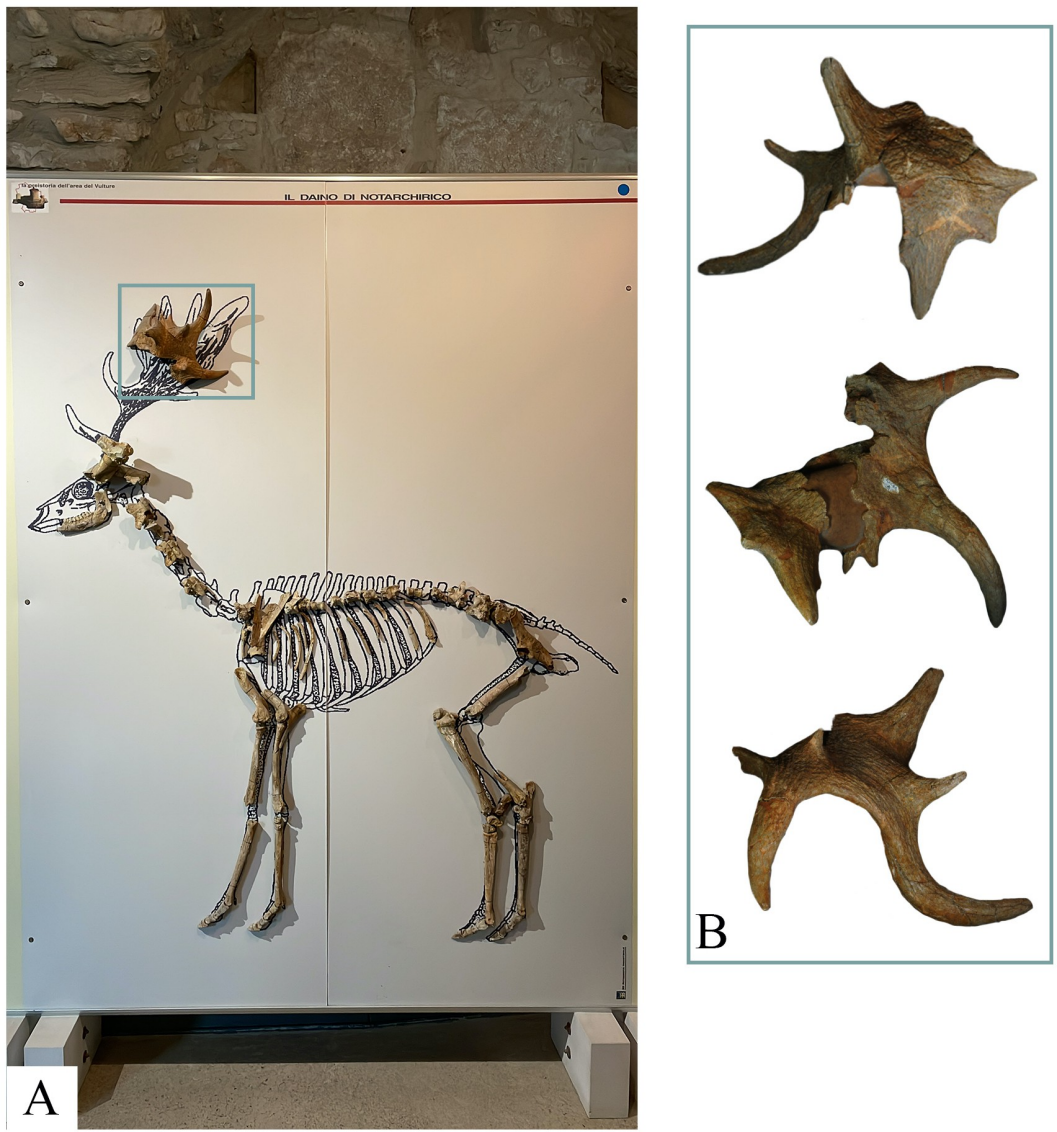
Composite skeleton of fallow deer currently exposed at National Archeological Museum “Mario Torelli” of Venosa (A). Distal portion of the antler (specimen 1860) ascribed here to *Cervus elaphus* (B). Image allowed by the agreement of the Italian Ministry of Culture and the Museo Parco Archeologico Melfi e Venosa.

In order to exhibit the findings from Notarchirico to the general public, a composite skeleton, comprising fossils recovered from A, α and above α, has been exhibited at the National Archeological Museum of Venosa “Mario Torelli” (Fig 10). This fossil was referred to *Dama* cf. *clactoniana* by [22]. During the revision of the skeleton, we confirm that all the bones belong to fallow deer, except the antler.

The Authors [22] remarked the three-dimensional aspect of the specimen with the presence of several tines with a circular section, rather than being flat as generally observed in the genus *Dama*. The distal portion of the antlers of *Dama clactoniana* is generally medio-laterally flattened with three/four long tines elongated upwards [79, 80].

**Fig 11:**
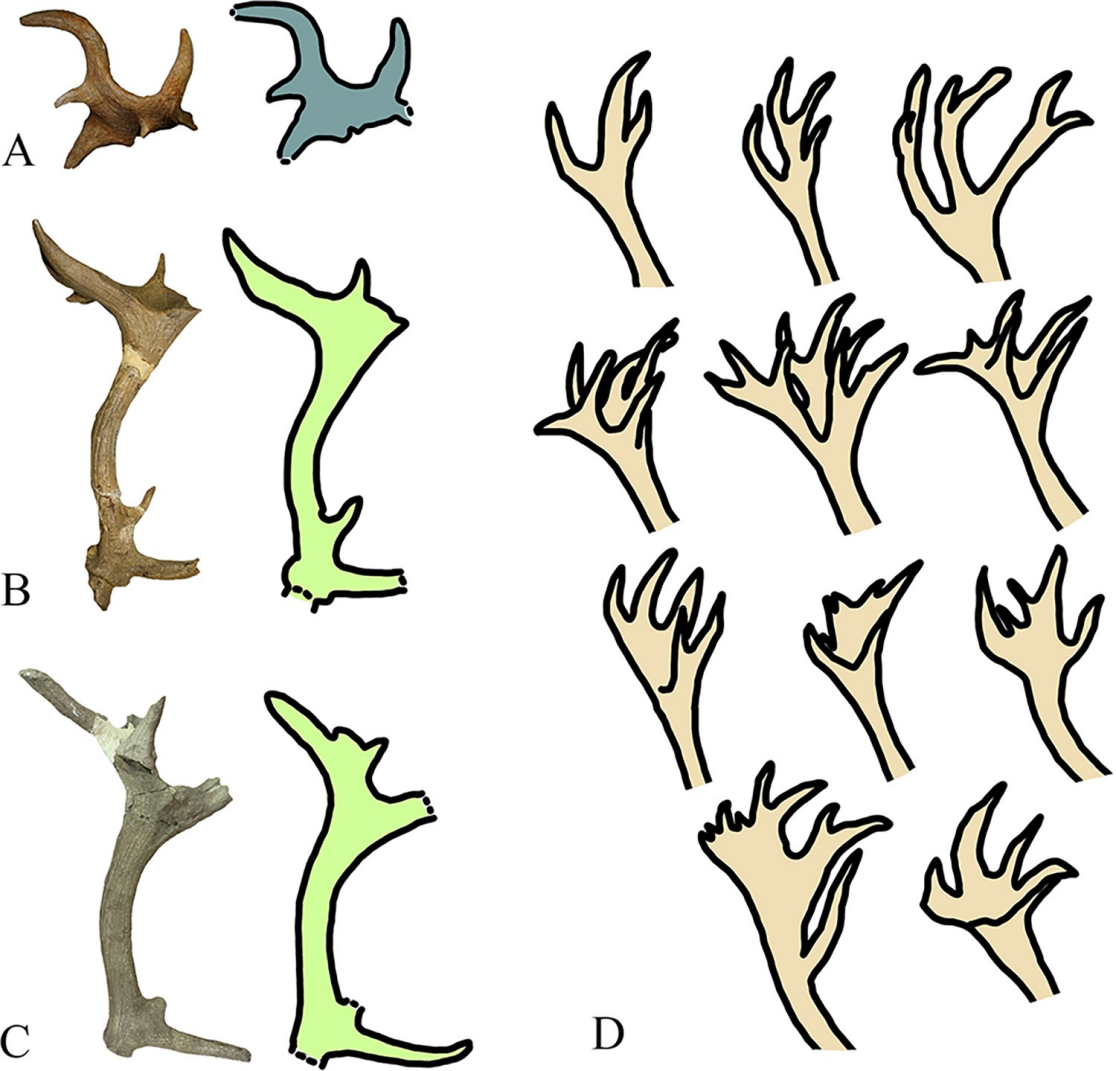
Antlers of fossil *Cervus elaphus* from Notarchirico (A), Ponte Molle (B) and Cava Nera Molinario (C). Variation observed in extant European populations of *Cervus elaphus* (modified by Castelli, 1941). Images are not in scale. Image allowed by the agreement of the Italian Ministry of Culture and the Museo Parco Archeologico Melfi e Venosa.

Our analysis confirms the skeletal attribution of the specimen to a portion of antler, characterized by a central flat portion surrounded by the presence of two well-developed tines and five reduced tines, but we assigned it to the terminal portion (crown) of *Cervus elaphus*. Indeed, the three-dimensional arrangement of the distal antler is observed in fossil and extant red deer [81–83]. The antler of red deer typically consists of three points on the beam, the brow tine, the bez tine and the trez tine, and a crown in the terminal part. This arrangement is generally reached at the 5^th^ or 6^th^ growth season. Over time, the antlers can become more complicated, with crowns that can have more than 3 points (but always ramified), a cup-shaped form, or a mix of these arrangements (ramifications with multiple palmated portions) [84] (Fig 11). Although antlers play a significant role in the taxonomic identification of Quaternary cervids, they are affected by individual and age variation. In order to better understand the morphological variability of antlers in red deer, 196 extant specimens (392 antlers) from Europe currently stored at the Hungarian Natural History Museum of Budapest (Hungary) have been analyzed. This sample highlights a huge variation in red deer antlers, with 97 individuals displaying differences between the right and left antlers. The development of terminal part is also highly variable: 79 antlers end with a simple fork (5 points), 123 with a true crown (6 or more points, but always ramified), and 40 with a cup-shaped arrangement, while the others display more complicated crowns or belong to juvenile individuals. The Author [84] also illustrated atypical morphotypes (antlers with more than 10 points) observed in extant *Cervus elaphus*. The author stated that these morphologies can be related to climatic factors, feeding availability, and health of the animal that affected the animal during the seasonal growth. The morphological features of the specimen 1860 resembles the cup-shaped crown observed in extant red deer ([84], and our observations). In the Italian Peninsula, similar crown arrangement was found at Ponte Molle and Cava Nera Molinario [81, 83] (Fig 11).

The fallow deer is the best represented taxon at Notarchirico, especially in the upper levels of the sequence. The sample from Notarchirico was attributed to *Dama clactoniana* by [22] and was initially considered one of the relevant taxa for biochronological attribution to mid Middle Pleistocene, but the specific attribution was mainly supported by the identification of the “aberrant” antler, which is revised herein as belonging to *Cervus elaphus*.

**Fig 12:**
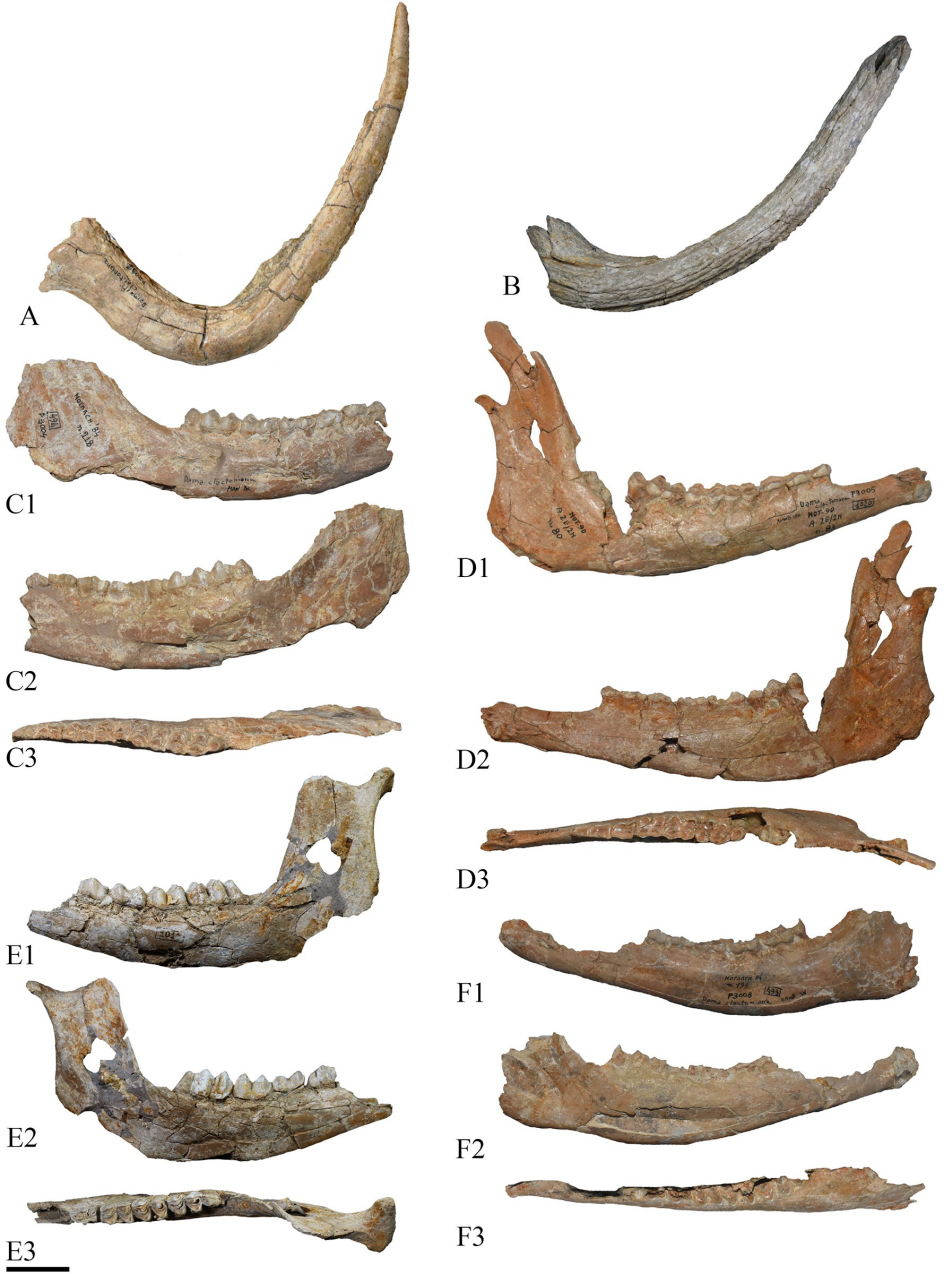
Fossil remains of *Dama* cf. *roberti* from Notarchirico. A–P2932, a right first tine of antler from the level α in lateral view; B– 378, a left first tine of antler from the level A in lateral view; C–P3004, a right hemimandible from the level A in labial (C1), lingual (C2) and occlusal (C3) views; D–P3005, a right hemimandible from the level A in labial (D1), lingual (D2) and occlusal (D3) views; E– 1012, a left hemimandible from the level A in labial (E1), lingual (E2) and occlusal (E3) views; F–P3008, a left hemimandible from the level A in labial (F1), lingual (F2) and occlusal (F3) views. Scale bar 3 cm. Image allowed by the agreement of the Italian Ministry of Culture and the Museo Parco Archeologico Melfi e Venosa.

The sample of the specimens truly referable to fallow deer primarily consists of isolated dental remains and postcranial bones, but also includes several nearly complete hemimandible and few antlers (Fig 12). Among the antler remains, the P2932 is one of the most informative fossils, representing a complete first (basal) tine broken at its base, at the level of its fusion with the beam (Fig 12). The specimen is very long and robust, and slightly compressed medio-laterally. Initially, it is directed anteriorly, but then turns upwards. These morphologies are generally observed in ‘*Pseudodama’* and *Dama roberti* [85], while the first tine is generally short, less robust, and anteriorly directed in *Dama clactoniana* [79, 80].

Dental remains from the upper levels of Notarchirico display a limited variability (Table 5). Some of these features are observed in *Dama clactoniana* but also in *Dama roberti* [80], and the presence of archaic dental features (the presence of the additional stylid between hypoconid and talonid in M_3_ and the common absence of the step between the lingual walls of the 2^nd^ and 3^rd^ lobes in M_3_) would be indicative of *Dama roberti* [78, 85].

**Table 5:** Dental features of *Dama* cf. *roberti* from the levels A–above α of Notarchirico.

Tooth	Dental features		Number of specimens
P_3_	Entoconid	Weakly angled to the transverse axis of the tooth	0
		Perpendicular to the transverse axis of the tooth	2
P_4_	Anterior hypoconid wing	Present and connected to the entoconid	1
		Absent	1
	Entoconid	Weakly angled to the transverse axis of the tooth	1
		Perpendicular to the transverse axis of the tooth	1
	Molarization	Present	3
		Absent	0
Lower molars	Ectostylids	Equally developed	5
		M_2_ larger than M_1_	0
	Mesial cingulum	Weakly developed	4
		Largely developed	0
M_3_	Step between the lingual walls of 2nd and 3rd lobes	Present	2
		Absent	4
	Ecostylid	Present	7
		Absent	0
	Additional stylid between hypoconid and talonid	Present	5
		Absent	2

During the new fieldwork, three remains of potentially belonging to this group have been collected for the first time from the lower levels of the sequence. Nevertheless, these only consist of a fragmentary upper molar from level I1 and a fragment of metatarsal and a third phalange from level G, which are of poor taxonomic value and hence attributed herein simply to *Dama*-like deer.

#### Cercopithecidae

This group is represented by two remains recovered from level G (Fig 5). The first, B24GNC, is a proximal portion of a well-preserved right ulna of *Macaca* sp., described by [34]. During the 2022 fieldwork, an isolated tooth was found, identified as a right deciduous upper canine. The tooth is very worn with the crown preserved few millimeters above the collar. The root is short and robust, and the crown is buccolingually compressed with a subtriangular shape in occlusal view (slightly expanded lingually). The general morphology of the deciduous upper canine is close to that of teeth of *Macaca sylvanus florentina* from Quibas [86] and Vallparadìs Estació [87].

#### Felidae

During surveys on the *in situ* archeo-paleosurfaces at Paleolithic Park of Notarchirico excavated until the 1990s, a lion remain was identified in the level A in the trench SI2 [33]. The fossil was temporarily removed by the surface and cleaned before the study and relocated in its original position later. Morphological and biometric analysis of NOT A 5N-9/10E 109 revealed its attribution to *Panthera spelaea* [33].

#### Canidae

In addition to partial humerus found in the cut 19 of Chiappella [37], the wolf remains recovered in the levels I2, I1 and G consist of isolated dental remains, and a first phalange (Fig 5).

Two fragments of lower first molar were found in the level I2, one preserves only the portion of trigonid (F36NC1) and the second with only the portion of the protoconid-talonid (E37NC2). F36NC1 has a large paraconid with a mesial margin that is slightly inclined distally. The protoconid is high and robust, while the metaconid is quite circular in shape, robust and located near the lingual side of the protoconid. E37NC2 shows a high and robust protoconid, with a circular in shape and robust metaconid along the lingual side of the tooth. The talonid basin is shallow, with a large and high hypoconid, a small entoconid, three cuspid-like on the distal cingulum and a small cuspid between the metaconid and the entoconid. These features are commonly observed in *Canis mosbachensis* and *Canis lupus* [88–90].

**Fig 13:**
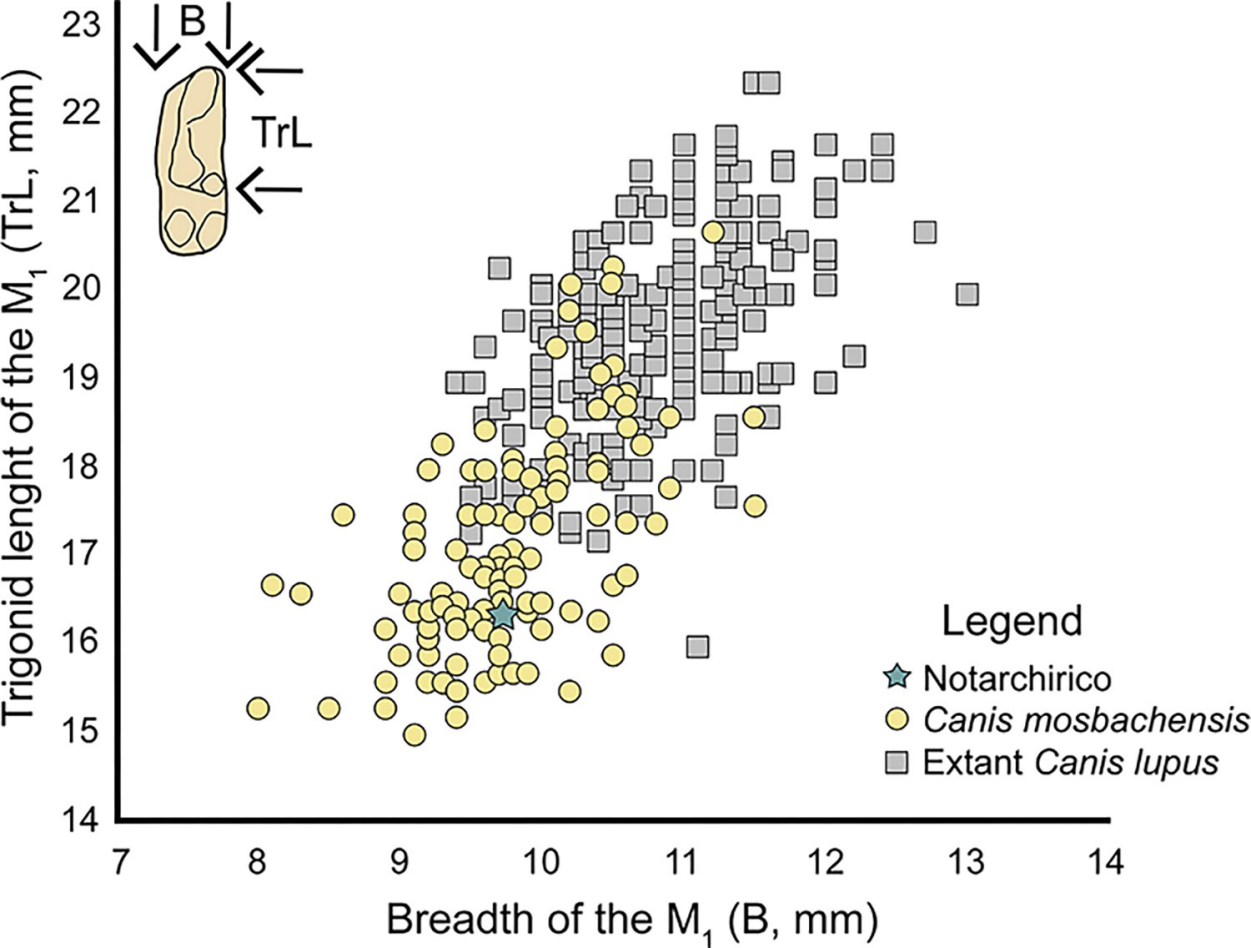
Plot of the trigonid length vs. the breadth of the lower first molar (M_1_) of *Canis mosbachensis* and extant *Canis lupus*.

The plot of the length of the trigonid *versus* the breadth of the lower first molar shows that the specimen from Notarchirico falls in the range of variation of *Canis mosbachensis* (Fig 13).

#### Mustelidae

This group is quite rare at Notarchirico, represented by an isolated fossil from level I1 and one in level H (Fig 5). Both remains are fragments of ulna, the first, B27NC1 is a proximal portion of right ulna with the olecranon disarticulated, while the second, E36NC2 is a proximal portion of left ulna, broken just above the trochlear notch. The two specimens display the anconeal process more developed anteriorly than the margin of the lateral and medial articular facets of the radius in lateral and medial views. The profile of the trochlear notch is semi-oval in shape, elongated proximo-distally. In anterior view, the medial articular facet of the radius is larger than the lateral one, and it is slightly inclined distally. These morphologies, in addition to the small dimension of the remains, are indicative of *Martes*, but there are no features for a specific identification.

#### Castoridae

A lower third molar was found in the level E during the historical excavation (Fig 5). This tooth is rectangular in shape in occlusal view, buccolingually compressed. It displays four lophs, anterolophid, metalophid, mesolophid and posterolophid. It also possesses four flexids, three on the lingual side, paraflexid, mesoflexid and metaflexid, and one in the labial side, the hypoflexid. The distal margin is slightly convex. These morphologies suggest the presence of *Castor fiber* [59].

**Fig 14:**
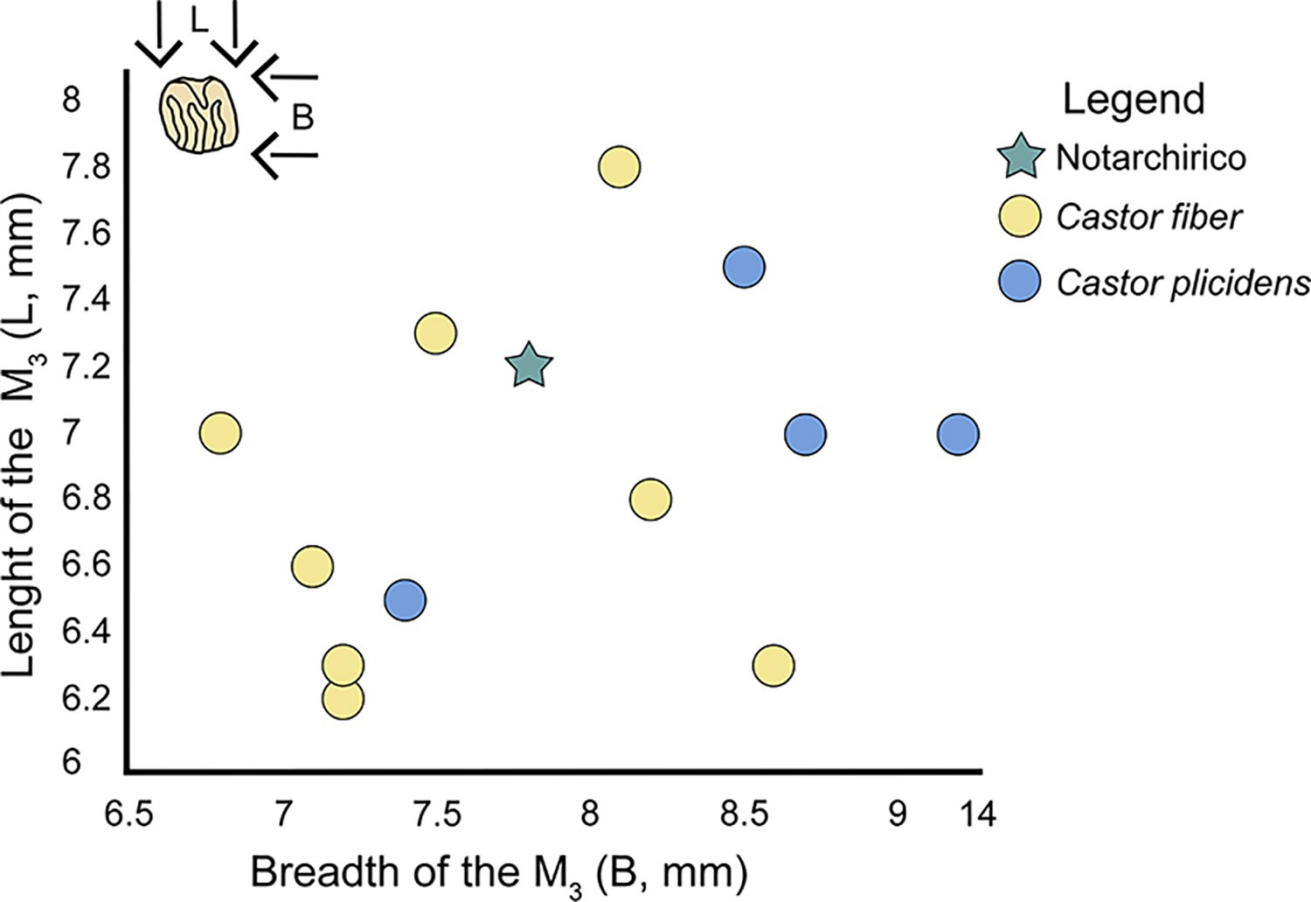
Plot of the length vs breadth of the lower third molar (M_3_) of fossil *Castor plicidens* and *Castor fiber*.

The biometric comparison of fossil and extant lower third molar (M_3_) of *Castor* shows a weak separation between the species (Fig 14), while the Notarchirico tooth occupy the central part of the graph.

During the new excavations, a first phalange, C35-34 was found in the level I1, documenting for the first time the presence of this species in the lower levels of the deposit.

## Discussion

### The faunal assemblages from Notarchirico

Frequentation of the site by Middle Pleistocene hominins, indirectly identified through the stone tools and some signs of consumption, was attested in each level of the succession [16, 29]. This prolonged and repeated frequentation of the site could indicate relatively good paleoenvironmental and paleoclimatic conditions for the end of MIS 17, and later a sort of a refuge area for hominin groups during the major climatic crisis of MIS 16, and as a possible starting point for re-peopling northern Europe during the earliest phases of the MIS 15. However, if the lithic tools have been collected in all sequence [16, 20, 29, 31, 32], the human-faunal interactions are difficult to establish, as consequence of the poor preservation of bone surfaces [16, 33, 35]. The putative exploitation of animal carcasses seems proven only for some middle and large-sized ungulates [16]. Geological and sedimentological analyses of Notarchirico indicated the presence of alluvial environments, with pebble and cobble deposits interpretated as lakeshores bed (I2, F, D, C, B and A), and sandy and clayey sediments filling water channels, indicting low-energy fluvial sedimentation [20]. Hominins likely inhabited the surroundings of Notarchirico, but these paleochannels offer at these groups water, wood and plant resources, as well as small chert nodules and limestone cobbles. It is reasonable to hypothesize that hominins could have an easy primary access to numerous ungulates who lived in the surrounding area, and who usually visited for access to fresh water and vegetation. Unfortunately, the generally bad preservation of bone surfaces makes difficult to investigate this topic [16]. Taphonomic analyses of the level α, albeit limited in scope, suggested as the faunal remains resulted accumulated by different agents, with the majority of mammals has died naturally in the surroundings of these paleochannels, and later have been secondarily transported, sorted, and modified by fluvial transport and trampling. Therefore, although the human frequentation of the site is quite constant all along the sequence, the faunal accumulation seems deposited by natural factors, without an intense selection by hominins, who probably acted as modification agents rather than accumulation ones. However, the archeozoological review of all fossil remains from Notarchirico is in progress and the hominin contribution to the fauna should remain open.

The extensive sedimentary sequence of Notarchirico preserves a rich fossil sample collected from various stratigraphic levels. Based on our results, three main complexes are identified (Fig 4): the lower complex (levels I2-G), the middle complex (levels F-C) and the upper complex (levels B-above α).

The differences in the number of remains and mammal species identified across the complexes could be related to the sediment volume excavated in both old and new excavations. Nevertheless, level C was investigated over 20 m^2^, level D over 25 m^2^, and level E over 40 m^2^ (Table 1). These levels, along with level H, contain the fewest mammal remains in the sequence. Conversely, one of the least investigated levels, level I (18 m^2^), is among the richest in fossils (162; Fig 3). This clearly indicates that the sediment volume excavated at Notarchirico did not influence the relative richness of fossils per level or their abundance across the different stratigraphic complexes.

The faunal assemblage from the lower complex (levels I2-G) includes *Palaeoloxodon antiquus*, *Stephanorhinus* sp. *Hippopotamus* sp. *Bison schoetensacki*, *Praemegaceros* sp., *Cervus elaphus*, *Dama-*like deer, *Macaca* sp., *Canis* sp., *Martes* sp. and *Castor fiber*.

*Palaeoloxodon antiquus* exhibited a mixed feeding diet, favoring forested areas where it consumed leaves, branches and soft grass [91–93]. Members of the genus *Stephanorhinus* are thought to have predominantly browse-based diets, indicating therefore the presence of forested landscapes [94]. Bovids were generally adapted to grasslands, showing mixed feeders or grass-dominated mixed feeders [93, 95]. Cervids mainly occupied dense forests, woodlands and shrublands [94]. Quaternary *Dama* were considered browsers, typically inhabiting forested landscape [17, 96–98]. *Cervus elaphus* can thrive in different habitats, preferring deciduous woodlands, mixed deciduous-coniferous, coniferous woodlands and Mediterranean maquis scrub [99–101]. Hippopotamus fossils are significant paleoenvironmental indicators, their presence strongly suggests humid conditions, mild winters, and the presence of water bodies as lakes, ponds or rivers during Quaternary [69 and reference therein]. The presence of macaque indicates shrublands to evergreen forests, with trees essential for sleeping, feeding, and escaping from predators [102, 103]. Eurasian beavers show a semi-aquatic life, generally inhabit freshwater habitats surrounded by woodland [104, 105]. It needs to mix of running and still waters, never completely frozen nor completely dry [106–108]. The mammal assemblage indicates the dominance of wooded spaces, sparse steppes, and the existence of water bodies (lakes or ponds), indicating a deterioration of the fully interglacial conditions recorded during the end of MIS 17.

The middle complex (levels F-C) is the poorest in number of mammals remains (Table 3). These levels documented the presence of *Palaeoloxodon antiquus*, *Bison schoetensacki*, *Praemegaceros* sp., *Cervus elaphus*, *Dama-*like deer and *Castor fiber* (Table 3). The relative abundance of straight-tusked elephant and bovine fossils in the middle complex suggests the presence of open space, while the relatively scarcity of cervids supports the hypothesis the limited forest development (Fig 3). In the middle complex of Notarchirico, species adapted to warm-temperate climatic conditions, such as macaques and hippopotamuses, disappear. The MIS 16, in general, is considered one of the most extreme glacials of the Pleistocene suggesting the extensive presence of open landscapes [6]. By integrating the age of these levels, the low number of mammal remains and their paleoenvironmental indications, the middle complex can be attributed to the glacial conditions of MIS 16 (Figs 3 and 4).

The faunal assemblage from the upper complex (from B to above α) includes *Palaeoloxodon antiquus*, *Sus scrofa*, *Bison schoetensacki*, *Praemegaceros* sp., *Cervus elaphus*, *Dama* cf. *roberti*, and *Panthera spelaea*. Similar to *Dama-*like deer, *D*. cf. *roberti* can be considered a browser and, therefore, an indicator of wooded areas [17, 96–98]. Modern European populations of *Sus scrofa* generally occupy forest areas, as evergreen oak forests, but also open spaces such as steppe and Mediterranean shrubland. The mammal assemblage from the upper complex is dominated by cervids (more than 75% of the remains), strongly suggesting that the landscape was dominated by forests. These levels indicate an improvement in climate, transitioning towards the full interglacial conditions of the of MIS 15.

### Biochronological implications

The evolution of European mammal paleocommunities was intimately linked to climatic oscillations occurred during the end of Early Pleistocene and the early Middle Pleistocene [7, 10, 15, 109, 110]. The EMPT records multiple dispersal waves that drove a significant reorganization of large mammal faunas, with a gradual disappearance of the last surviving Villafranchian taxa [7, 110, 111]. Mammal dispersal included species arriving from Asia and Africa, as documented by Asian newcomers like *Cervus elaphus* and *Sus scrofa* [4, 8, 110, 112, 113].

An important proposal made by Cassoli et al. [22] was the introduction of Notarchirico as a FU which would have occupied a position intermediate between the Isernia (older) and Fontana Ranuccio (younger) FUs. When Cassoli et al. [22] introduced this hypothesis, Isernia La Pineta was dated ca. 700 ka, but its chronology was later revised by [114], indicating an age of approximately 610 ka. The last chronostratigraphical reassessment of Isernia La Pineta was carried out by [115], who constrained the deposit between ca. 587 ka and 583 ka. Therefore, the two localities became of similar age and the fauna of Notarchirico was considered a local fauna of the Isernia FU [23–25].

However, uncertainty persisted on the biochronological attribution of the upper levels of Notarchirico (A and α), since [22] reported the presence of *Bos primigenius* and *Dama clactoniana*, whose earliest dispersals are generally placed within the Fontana Ranuccio FU (e.g., [69, 75, 85, 113]).

Cassoli et al. (1999) identified *Bison* sp., *Bos primigenius*, Bovinae cfr. *Bos primigenius* and Bovinae indet from levels A and α of Notarchirico. These remains were revised in this work and mostly ascribed to *Bison schoetensacki*, while badly preserved and fragmentary specimens were referred to as Bovinae indet. By excluding *Bos primigenius* from Notarchirico, the oldest clear evidence of this species come from Ponte Molle (MIS 13, [83]), Fontana Ranuccio (MIS 11, [116]), Fontignano 2 (MIS 11, [113]), Malagrotta (MIS 11, [55, 117]) and Castel di Guido (MIS 11, [117, 118]). The record of *Bison schoetensacki* from the upper levels of Notarchirico is one of the latest occurrences of this taxon in Europe, which disappear after the Isernia FU [43, 71].

One of the most iconic records of *Dama clactoniana* in Europe was from Notarchirico, where a composite skeleton comprising fossils recovered from upper levels (A, α and above α) was exposed at the National Archeological Museum of Venosa “Mario Torelli” (Fig 10). The review of these fossils, in addition to others recovered in the site but not exposed in the Museum, suggests that these remains can be ascribed to *Dama* cf. *roberti*. Indeed, the most important element for the identification of *Dama clactoniana* at Notarchirico was the upper part of the antler (exposed at National Archeological Museum of Venosa “Mario Torelli”), which, as discussed above, has a morphology that best fits with an attribution to a cup-shaped crown of *Cervus elaphus* ([84]; Fig 11). *Dama roberti* is a taxon of established only in 2013 (not yet known when the Notarchirico fallow deer was initially described in 1999) which is recognized in Europe from the early Middle Pleistocene, as documented in the sites of Pakefiled (0.7 Ma; [119, 120]), West Runton (700–600 ka; [119]) and Soleilhac (700–600 Ma; [121]). Italian samples tentatively attributed to this species were collected from Cesi, Contrada Monticelli, Isernia La Pineta and Valdemino cave [75, 80, 122, 123]. By disproving the presence of *Dama clactoniana* at Notarchirico, the hypothesis of its spread in Europe during the mid-Middle Pleistocene (MIS 13) would be confirmed, as suggested by the records of Ponte Molle, Tor di Quinto, and Visogliano ([80] and reference therein).

**Fig 15:**
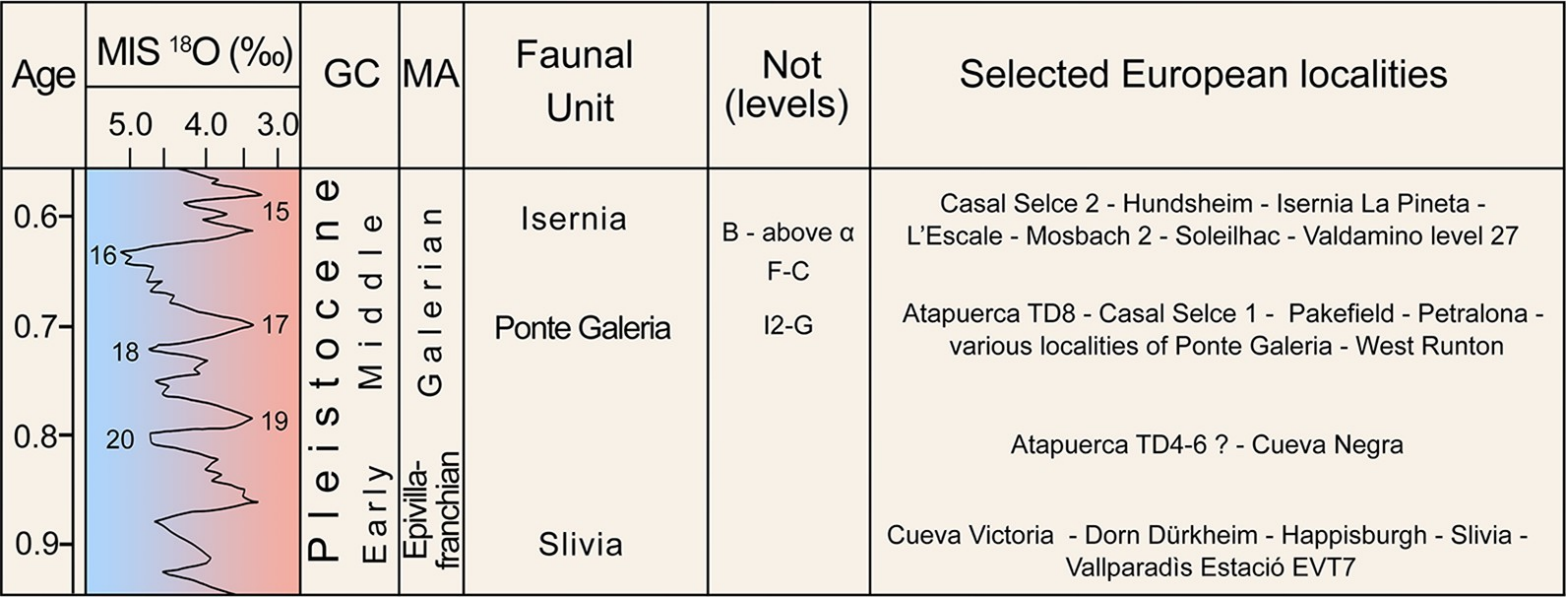
Quaternary time scale, biochronological scheme with the three complexes of Notarchirico and selected European localities. Question marks denote uncertain attributions. Abbreviations: MIS Marine Isotope Stage; GC–—Geochronology; MA–Mammal Ages; Not–Notarchirico.

However, the extensive paleontological record of Notarchirico in its firm chronological context assumes an important role for the biochronology of European land mammals (Fig 15).

All sequence yielded remains of *Palaeoloxodon antiquus*, one of the most common species of the European paleocommunities over the last 800 ka. The earliest dispersal of the straight-tusked elephant is still a controversial topic. The putative presence of this taxon in the European Early Pleistocene was reported from Spain localities of Vallparadís Estació [124] and Huescar [125]. These records have been later revised and referred to as *Mammuthus* lineage [126–128]. Fragmentary remains of proboscideans from latest Early Pleistocene site of Slivia (Italy) described by Bon et al. [129] were attributed to *Palaeoloxodon* cf. *antiquus*, later considered as *Palaeoloxodon antiquus* by Palombo & Ferretti [130]. The record from Slivia could represent the earliest arrival of this species in Europe [131]. Fossils of straight-tusked elephant improved during the Middle Pleistocene, being found in all European continent [131–133]. In this biochronological context, the well-dated record of Notarchirico confirms the presence of *P*. *antiquus* in southern-western Europe during the early Middle Pleistocene.

The dispersal of *Cervus elaphus* took place during the latest Early Pleistocene [4, 10, 15, 134]. The oldest fossils of *Cervus elaphus* are from the levels TD6 of Gran Dolina (Spain, [15], Dorn Dürkheim (Germany, [135]) and HSB3 of Happisburgh (Britain, [120]). In Italy, oldest fossils should be those of Slivia, attributed to *Cervus* sp. by Bon et al. [129]. The sample of Notarchirico represents one of the earliest *Cervus elaphus* in Italian Peninsula.

Pleistocene lions are quite scarce in the European record, affecting the reconstruction of the evolutionary history of this large cat. Attested fossils of lion were found at Kozi Grzbiet (Poland, ca. 750–700 ka; [136]); and Pakefield and West Runton (England, ca. 700–600 ka; [137]). The existence of large-sized pantherine remains has been reported from some latest Early Pleistocene sites in the Iberian Peninsula (Barranc de la Boella, Cueva Victoria and Vallparadís Estació; [138]), but these need to be deeply described. Recently, the fragmentary IV metatarsal, recognized in the level A in the SI2 trench during surveys on the archaeo-palaeosurfaces excavated until the 1990s musealised *in situ*, was referred to as *Panthera spelaea* [33]. This finding is the oldest occurrence in southwestern Europe of the cave lion known to date.

Remains of Eurasian beavers are relatively scarce in the fossil record, probably due to their limited ecological tolerance. Oldest specimens come from the Early Pleistocene Spanish site of Sima del Elefante (1.4–1.2 Ma; [139, 140]) and TD5- TD6 levels of Gran Dolina [139–141]. The record of Notarchirico represents the earliest attested record of this taxon in Italian Peninsula, also proving its presence in southern Mediterranean Europe.

## Conclusions

Following the revision of the paleontological sample of Notarchirico carried out herein, three different mammal complexes are recognized, documenting the response of the fauna to the climatically driven changes occurred between approximately 700 ka and 600 ka. The lower complex (levels I2-G) records extensive forested areas and warm-temperate conditions during the end of MIS 17; the middle complex (levels F-C) is the poorest in terms of number remains and suggests the dominance of open spaces and full glacial conditions of MIS 16; the upper complex (levels B-above α) is the richest for mammal remains and marked the massive return of wooded landscape and, in general, of improvement in climate, towards the full interglacial conditions of MIS 15.

Biochronologically speaking, Notarchirico documents one of the earliest occurrences in Europe of *Palaeoloxodon antiquus*, *Cervus elaphus*, *Dama* cf. *roberti* and *Panthera spelaea*, and one of the last records of *Bison schoetensacki*. In addition, the putative presence of *Bos primigenius* and *Dama clactoniana* in the upper levels (A and α) reported in previous works is not validate here.

Our results confirm the importance of the review of historical museum collections, which can offer new paleontological data for understanding the impact of climatic changes on past terrestrial ecosystems. In addition, the both revised and new data reported here provide valuable information on faunal changes over the EMPT in Mediterranean Europe.

## Supporting information

S1 Table Measurements of *Bison schoetensacki*, *Canis* sp. and *Castor fiber* from Notarchirico. (XLSX)
